# Pesticide prioritization based on risk and model diet proposals for assessing cumulative exposure to pesticide residues in the Brazilian population through food consumption

**DOI:** 10.1002/ps.70507

**Published:** 2026-01-13

**Authors:** Bianca Figueiredo de Mendonça Pereira, Bernardete Ferraz Spisso

**Affiliations:** ^1^ Programa de Pós‐Graduação em Vigilância Sanitária, Instituto Nacional de Controle de Qualidade em Saúde, Fundação Oswaldo Cruz Rio de Janeiro Brazil; ^2^ Departamento de Química Instituto Nacional de Controle de Qualidade em Saúde, Fundação Oswaldo Cruz Rio de Janeiro Brazil

**Keywords:** chemical compound classification, chemical hazard, exposure assessment, food safety, risk ranking, risk prioritization

## Abstract

**BACKGROUND:**

Pesticide residue exposure assessments can be carried out during both the pre‐regulation and post‐regulation phases of these substances. Due to a lack of consumption data required for these assessments, model diets have emerged as a practical solution. However, diets that more accurately reflect each country's consumption patterns provide a more reliable basis for calculating long‐term residue exposures and verifying established maximum residue limits. The definition of model diets based on a specific population allows for the prioritization of matrices for analysis and, consequently, monitoring program optimization, due to a more rigorous selection of food groups that may comprise residue exposure sources. This study aimed to assess the contribution of foods to cumulative priority pesticide residue exposure and propose model diets that reflect Brazilian food consumption patterns.

**RESULTS:**

The present study considered a chemical compound risk classification and a sensitivity analysis to identify the foods that contribute most to pesticide residue exposure. The top five pesticide–crop combinations in the risk ranking were chlorpyrifos in citrus, banana and potato (score 168) and (2,4‐dichlorophenoxy)acetic acid (2,4‐D and methomyl in rice (score 140). Model diets were developed based on Brazilian dietary habits in accordance with exposure assessment principles and included the 22 foods considered most critical regarding pesticide exposure.

**CONCLUSION:**

A practical simulation using selected pesticides demonstrated that conventional model diets may not yield the same results as the specific model diets proposed for Brazil. © 2026 The Author(s). *Pest Management Science* published by John Wiley & Sons Ltd on behalf of Society of Chemical Industry.

## Graphical abstract

A risk classification of registered pesticides in Brazil was conducted, and the contribution of food to cumulative pesticide exposure was evaluated. Model diets were proposed, based on Brazilian consumption patterns.

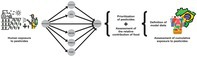

## 1 INTRODUCTION

Global pesticide use increased by 70% from 2000 to 2022, with the highest consumption recorded in the Americas, followed by Asia, Europe, Africa, and Oceania. In 2022, pesticide application rates were also highest in the Americas, followed by Oceania, Asia, Europe, and Africa. That same year, Brazil had the highest pesticide use per cultivated area (12.6 kg ha^−1^) compared to other countries.^1^

The use of these substances can result in pesticide residues in targeted foodstuffs. Sources of exposure vary, including direct contact by farmers who handle and apply these products to crops, as well as the ingestion of animal‐derived food products exposed to pesticides. Additionally, pesticides can contaminate various environmental compartments, such as soil, air, and water.^2^, ^3^ Exposure to pesticide residues is, in fact, a significant concern because these substances are designed to interfere with natural biological processes and are therefore toxic and potentially harmful to human health.^4^

The acceptable daily intake (ADI) estimates the amount of pesticide residue (mg per kg of body weight) that can be ingested daily over a lifetime without health risks, based on available data at the time of assessment. The acute reference dose (ARfD) represents the amount of a pesticide that can be consumed within 24 h without significant health risks, also expressed in mg per kg of body weight.^5^ The maximum residue limit (MRL) in food is the highest concentration of a pesticide residue legally permitted, and foods complying with MRL are considered toxicologically acceptable for consumer health.^6^

In Brazil, studies on pesticide residues in plant‐based products, which support the establishment of MRLs, focus on specific pesticides applied to designated crops for registration purposes. These studies include both field and laboratory phases. Field studies simulate the proper use of pesticides on crops, ensuring that the total applied amount aligns with agronomic efficacy tests and product leaflet guidelines. For processed foods, the MRLs established for natural foods apply, except when the food is dehydrated or concentrated, in which case specific methodologies from the Codex Alimentarius must be followed.^7^ Some regulatory agencies use statistical methods to harmonize MRL estimates derived from the same residue data but assessed by different evaluators. This approach can be applied using the calculator developed by the Organization for Economic Cooperation and Development.^8^

The risk associated with consuming food containing hazardous compound residues must be assessed to establish quality standards.^9^ This risk is determined by the correlation between a compound's toxicity and population exposure.^10^

Dietary risk assessments for pesticide residues in foodstuffs are conducted, among other instances, during pesticide registration to determine whether population exposure based on MRL values is acceptable. The risk is deemed unacceptable when dietary exposure calculations exceed guideline values.^11^ Additionally, pesticide residue exposure assessments must consider the potential co‐occurrence of multiple residues in a single foodstuff, with monitoring studies providing key data on this issue.^12^

The general principles and methods for chemical risk analysis applied by the Joint Meeting on Pesticide Residues (JMPR) guide pesticide residue exposure assessments within the Food and Agriculture Organization (FAO) and the World Health Organization (WHO).^9^, ^13^, ^14^ Dietary exposure assessment, a component of risk assessment, evaluates the relationship between the concentration of a certain chemical compound in a population's diet and the consumption of foods containing its residue. The outcome is expressed as chemical compound exposure estimates.^13^

Consumption data are essential in exposure assessments but are not always available. This has led to the creation of model diets. Cluster diets (regional model diets) were first developed by the WHO to estimate dietary exposure to radionuclides in food following the Chernobyl accident in 1989. Derived from the Food Balance Sheets of the FAO, they were representative of the dietary patterns of the Middle East, the Far East, Africa, Latin America, and Europe. In 1997, the WHO introduced the clustered consumption diets into the program known as the Global Environment Monitoring System (GEMS)/Food Contamination Monitoring and Assessment Programme, widely referred to as GEMS/Food, with the aim of estimating exposure to various chemicals present in food.^15^ The WHO employs data from GEMS/Food to carry out the exposure assessment step, considering several diets referred to as GEMS/Food cluster diets.^16^, ^17^ Exposure assessments should be carried out employing national data, whenever available. However, aggregated data are more suitable for comparing average consumption across countries, which is essential for risk assessments at the international level.^15^ The model diet approach is complementary to monitoring and surveillance programs because it provides a reliable basis for estimating the dietary exposure of a certain population to food chemicals, including residues, and their public health impacts.^18^, ^19^

However, although the use of model diets ensures coverage of highly consumed foods, there is no information regarding the contribution of a specific compound in risk assessment through food consumption because residue concentrations are not considered. In other words, highly consumed foods may not be the main source of exposure to a given residue.^20^ In addition, the human population is exposed to numerous chemical substances through different pathways and routes, with varying exposure times, which results in increased health risks compared to exposures to individual chemicals. The potential combined effects are expressed through similar or different mechanisms of these substances and should be verified through cumulative residue exposure assessments.^21^, ^22^ The combined toxicity of two or more pesticides to human health may result from dose addition, response addition, or interactions among those compounds. Dose addition occurs when substances act through the same mode of action, differing only in their potencies. In response addition, substances exhibit different and independent modes of action, nature, and toxic effect sites. Finally, interactions encompass all other forms of joint action, and the combined effect may be stronger (synergistic) or weaker (antagonistic) than expected based on dose or response addition.^23^ Cumulative risk assessments are conducted by combining exposures to different compounds according to their toxicities through the cumulative assessment groups (CAGs) definition. A CAG is a group composed of substances that share a specific common characteristic. Studies have been conducted to demonstrate the risk of cumulative exposure to specific CAGs. These include chronic cumulative risk assessment of dietary exposure to pesticides for thyroid‐related effects such as hypothyroidism, hypertrophy, hyperplasia, and C‐cell neoplasia; acute cumulative risk assessment of dietary exposure to pesticides for two nervous system outcomes, namely inhibition of brain and/or erythrocyte acetylcholinesterase and functional motor division alterations; cumulative risk assessment concerning chronic acetylcholinesterase inhibition; and cumulative risk assessment for two types of craniofacial alterations (changes due to abnormal skeletal development, soft tissue abnormalities of the head, and neural tube defects of the brain).^22^, ^24^, ^25^, ^26^, ^27^

In this context, this study aimed to propose model diets that reflect Brazilian food consumption patterns, optimizing long‐term cumulative pesticide exposure calculations and MRL verification. First, a risk classification was conducted for pesticides with active registrations in Brazil. Next, model diets were developed by assessing the contribution of *in natura* and minimally processed foods to cumulative pesticide exposure. Finally, the proposed model diet for Brazil was compared to group 5 of the GEMS/Food cluster diets in an exposure assessment.

## 2 MATERIAL AND METHODS

A pesticide classification was conducted to assess cumulative dietary pesticide residue exposure. Prioritization was based on the severity and probability of exposure, according to Eqn (1):

(1) Risk=severity×probability

A general risk classification based on the scoring method was employed to prioritize pesticides.^28^ This semiquantitative method uses scores to assess both the severity and likelihood of human health risks. Severity was represented by the ADI value score (Factor A) and by harmful health characteristics (Factor B). The ADI values were obtained from the Brazilian National Health Surveillance Agency (Anvisa, Brazil) website.^29^

Factor B was based on data from the WHO classification of acute pesticide risks to human health and the list of highly hazardous pesticides defined by the Pesticide Action Network (PAN).^30^, ^31^ The probability of exposure was scored using two independent factors (Factors C and D). Factor C was determined from the average *per capita* acquisition data on food consumption from the 2017/2018 Household Budget Survey (POF). The calculation was based on national acquisition data.^32^ The same reference values established for natural foods were applied to minimally processed foods. Occurrence data from monitoring programs on plant‐based foods by the Brazilian Ministry of Agriculture and Livestock (MAPA, Brazil) and Anvisa, as well as on water by the Ministry of Health, were used to assess Factor D. Additionally, a factor related to sales volume (Factor E) was incorporated in Factor D.^33^, ^34^, ^35^, ^36^, ^37^ The overall score for each substance was calculated by multiplying the coded values for severity, use, and residue occurrence. Eqn (2) was used to score the risks of currently registered pesticides.

(2) $$\text{Risk score}=\left(A+B\right)\;\times \;C\;\times \;\left(D+E\right)$$

Only pesticides with authorized agricultural use and current Brazilian registrations at the time of the study were included in the evaluation. Pesticides classified as biological or biochemical products were not evaluated.

Given the large number of active ingredients registered in Brazil, only pesticides with risk scores ≥10 were included in sensitivity studies to assess the contribution of each food to cumulative pesticide exposure. The sensitivity analysis was performed by calculating the relative contribution of each food, determined by the ratio of its exposure to the total average exposure from all foods (Eqn (3)). Additionally, the exposure contribution of each substance through food consumption was calculated relative to the health‐based guide value (HBGV), specifically the ADI, as shown in Eqn (4). The results were expressed as percentages, with calculations performed for each pesticide per food product.^20^ The average *per capita* food consumption data for the total national population and the five Brazilian macro‐regions, as described in the POF 2017–2018, were applied in Eqns (3) and (4). When such data were unavailable, average acquisition data were used.^32^, ^38^ For occurrence data, the MRLs established in Brazilian legislation were applied and substances without established MRLs were not considered.

(3) $$\text{Relative contribution}=100\;\times \;\frac{\text{average consumptio}n_{\text{food}}\left(\mathrm{mg}\;\mathrm{da}y^{-1}\right)\;\times \;\text{occurrenc}e_{\text{food}}\left(\mathrm{mg}\;kg^{-1}\right)}{\sum_{\text{Food}\;1}^{n}\text{average consumption}\;\times \;\text{frequency}}$$

(4) $$\text{Contribution of food}\;X\;\text{in relation to HBGV}\left(\%\right)\;=100\;\times \;\frac{\text{average consumptio}n_{\text{food}}\left(\mathrm{kg}\;\mathrm{da}y^{-1}\right)\;\times \;\text{occurrenc}e_{\text{food}}\left(\mathrm{mg}\;kg^{-1}\right)}{\text{HBGV}\left(\mathrm{ADI},\mathrm{mg}\;kg^{-1}\mathrm{bw}\right)\;\times \;60\;\mathrm{kg}}$$

Following Sieke, relative contributions of 10% or more were considered significant enough to alter total exposure estimates.^20^ Foods with contributions ≥10% of total daily consumption were included in the proposed model diets.

Additionally, a chronic cumulative exposure assessment was performed using a deterministic approach to estimate the theoretical maximum daily intake (TMDI) as per Eqn (5). The evaluation was carried out using the proposed model diet with average consumption data for the general Brazilian population and MRL values established in Brazilian legislation, with ADI values used for comparison with TMDIs. The results were compared using consumption data used in the model diet from group 5 of the GEMS/Food cluster diets for the TMDI calculation to assess pesticide exposure. A risk characterization was also conducted by calculating the hazard index (HI) (Eqn (6)).^11^, ^15^, ^38^

(5) $$\text{TMDI}=\frac{\sum \left(\mathrm{MRL}\left(\mathrm{mg}\;kg^{-1}\right)\;\times \;\text{average daily consumption}\;\mathrm{per}\;\text{capita}\left(\mathrm{kg}\right)\right)}{\text{body weight}\left(\mathrm{kg}\right)}$$

(6) $$\mathrm{HI}=\sum_{i=1}^{n}\mathrm{HQi},\text{where}\;HQ_{i}=\mathrm{Ex}p_{i}/RV_{i}$$

where HQ is the hazard quotient, Exp_*i*_ is the exposure of the individual chemicals in the mixture, and RV_*i*_ is the reference value of the individual chemical in the mixture (e.g. ADI).

Microsoft Excel® software was used for all calculations.

## 3 RESULTS AND DISCUSSION

### 3.1 Pesticide prioritization for assessing cumulative dietary residue exposure

Depending on the goal of the exposure assessment, human health impacts from dietary exposure to a single chemical substance residue, aggregate exposure (from multiple sources), or cumulative exposure to multiple substances may be evaluated.^18^, ^39^ While assessing dietary exposure to a single chemical is common, the human population is exposed to numerous substances from various sources. The combined effects of different pesticide residues may increase health risks because of similar or different mechanisms of action.^12^, ^21^

Cumulative exposure assessments involve combined exposure to multiple stressors (both chemical and non‐chemical) through various exposure routes.^40^ Highly toxic pesticides pose greater health risks compared to moderately toxic ones. However, higher risks arise from high exposure to moderately toxic pesticides than from low exposure to highly toxic ones.^2^ In food safety, prioritizing chemical substances for cumulative dietary exposure assessment is part of risk‐based monitoring, which considers both the probability and severity of exposure. This strategy helps detect potentially harmful agents that could cause significant adverse health effects. Moreover, defining monitoring priorities allows for more effective resource allocation.^28^, ^41^, ^42^

Semiquantitative methods are less complex, more informative, and commonly used to classify hazardous chemicals. By applying scores to assess severity and probability, these methods enable comparisons across a wide range of chemical agents.^28^ In this study, all pesticides registered for agricultural use, except for biological products, products with no associated crops, synthetic pheromones, some inorganic compounds, and compounds without an established ADI, were evaluated for prioritization using a risk matrix.^29^ A total of 6437 correlations between active ingredients and authorized crops were assessed (Supporting Information, Table S1).

The ADI value is a measure used to infer the potency of a substance. A higher ADI indicates a lower potential for adverse human health effects from ingestion through food, making it safer.^43^ Therefore, substances with lower ADI values received higher Factor A scores.

Factor B values, which refer to evidence of toxicological effects, were determined by the sum of pesticide classification values recommended by the WHO based on associated hazards, along with the scores from the international list of highly hazardous pesticides established by the PAN.^30^, ^31^ The WHO classification primarily relies on acute oral and dermal toxicity data from rats, with adjustments made when human hazards differ from those indicated by median lethal dose (LD50) assessments, i.e. the amount of a chemical that is lethal to one‐half (50%) of the experimental animals exposed to it. The PAN list defines highly hazardous pesticides based on four criteria: acute toxicity, chronic health effects, environmental risk, and international regulations. The final score results from the sum of individual scores across these criteria. A compound is included in the list if it scores in at least one of the groups).^30^, ^31^

The average values of national *per capita* food acquisition from crops registered for pesticide use were scored to determine exposure probability (Factor C).^32^ Additionally, detection evidence was scored based on the results from the following: (i) monitoring programs within the scope of the National Plan for the Control of Residues and Contaminants (PNCRC), conducted by MAPA from 2018 to 2020, (ii) non‐compliant results of pesticides with a detection percentage above 1% as reported by the Pesticide Residue Control Program (PARA) for the 2018, 2019, and 2022 cycles carried out by Anvisa, and (iii) results from the National Program for Surveillance of Water Quality for Human Consumption (Vigiagua) from 2020 to 2022, conducted by the Ministry of Health (Factor D). All non‐compliant pesticides from at least one report of each monitoring program published up to the date of this assessment were scored.^33^, ^34^, ^35^, ^36^

The best‐selling products were considered as Factor E, using data from the 2022 pesticide sales report published by the Instituto Brasileiro do Meio Ambiente e dos Recursos.^37^ The evaluated parameters were assigned scores ranging from 1 to 5, with the highest scores indicating the greatest risks. The values defined for the different factors are presented in Table 1.

**Table 1:** Scores assigned to the various pesticide risk factors

Score	Factor A: ADI (mg kg^−1^ bw per day)	Factor B: Toxicological classification		Factor C: *Per capita* household food acquisition per day (g)	Factor D: Occurrence in monitoring programs	Factor E: Sales in the country in 2022 (tons AI)
		WHO	PAN maximum score of the groups			
1	>10	Unlikely risk of acute effect	1	<5	Non‐compliant samples	≤500
2	>0.1–10	Slightly hazardous (III)	2	5 to <10	–	>500–2,500
3	>0.001–0.1	Moderately hazardous (II)	3	10 to <50	–	>2,500–10,000
4	≤0.001	Highly hazardous (Ib)	4	≥50	–	>10,000
5	–	Extremely hazardous (Ia)	–	–	–	–

ADI, acceptable daily intake; AI, active ingredient; bw, body weight; PAN, Pesticide Action Network; WHO, World Health Organization.

Employed compound classification, prioritization, and hierarchy approaches do not follow standardized techniques and depend on the specific study aim. In this context, methods for classifying chemical hazards such as pesticides and veterinary drugs remain limited. In this assessment, the risk classification system was adapted from a method developed in the UK for monitoring veterinary drug residues in food, based on available toxicological and probability data.^44^, ^45^

The Factor D score was limited to 1 point for samples with non‐compliant results because the occurrence data from the various assessments employed in this study indicated a low number of non‐compliant results, ranging from 0% to 3% of the total samples. Exceptions included pirimiphos‐methyl (6.4%) in the 2018–2019 PARA cycle, and acephate (5.1%) and formetanate (3.6%) in the 2022 cycle of the same program.^33^ The PNCRC in products of vegetal origin data could not be used as a scoring criterion for risk Factor D because the results are presented as percentages of compliant samples and the specific pesticide that exceeded the permitted limit for a given crop is not identified. Therefore, out of a total of 2715 samples analyzed during the 2018–2020 cycles, 335 non‐compliant results, ranging from 0.74% to 63.59% of the samples, could not be considered.^36^

The scores for the hazard factors of the evaluated compounds are provided in Supporting Information, Table S1. The results of the risk matrix ranged from 2 to 168 in total score, considering all evaluated pesticides.

Factors A, B, D, and E for each pesticide were scored independently of the associated crop, with the crop with the highest score being determined by consumption. Equal weight was applied to all factors in the calculation. However, it is important to note that the values can be adjusted by applying a weight value if one factor is deemed more significant than the others for classification.^28^ Both severity and probability were scored using quantitative data when available.

Pesticides with scores lower than 10 presented the following score variations: Factor A ranged from 1 to 4 points, with 80% of pesticides having a score of 3; Factor B ranged from 1 to 6, with 59% of pesticides having a score of 1; Factor C ranged from 1 to 4, with 89% of pesticides having a score of 1; Factor D had a score of 1 for 7% of pesticides; and Factor E ranged from 1 to 2, with 97% of pesticides having a score of 1. A value of 1 was assigned to Factor C for foods with no available consumption data, which is a limitation and source of uncertainty in the applied classification method.

**Figure 1:**
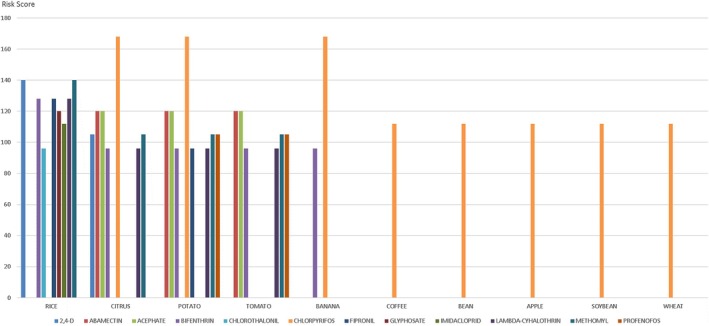
Highest‐scoring pesticides and their respective related crops.

**Figure 2:**
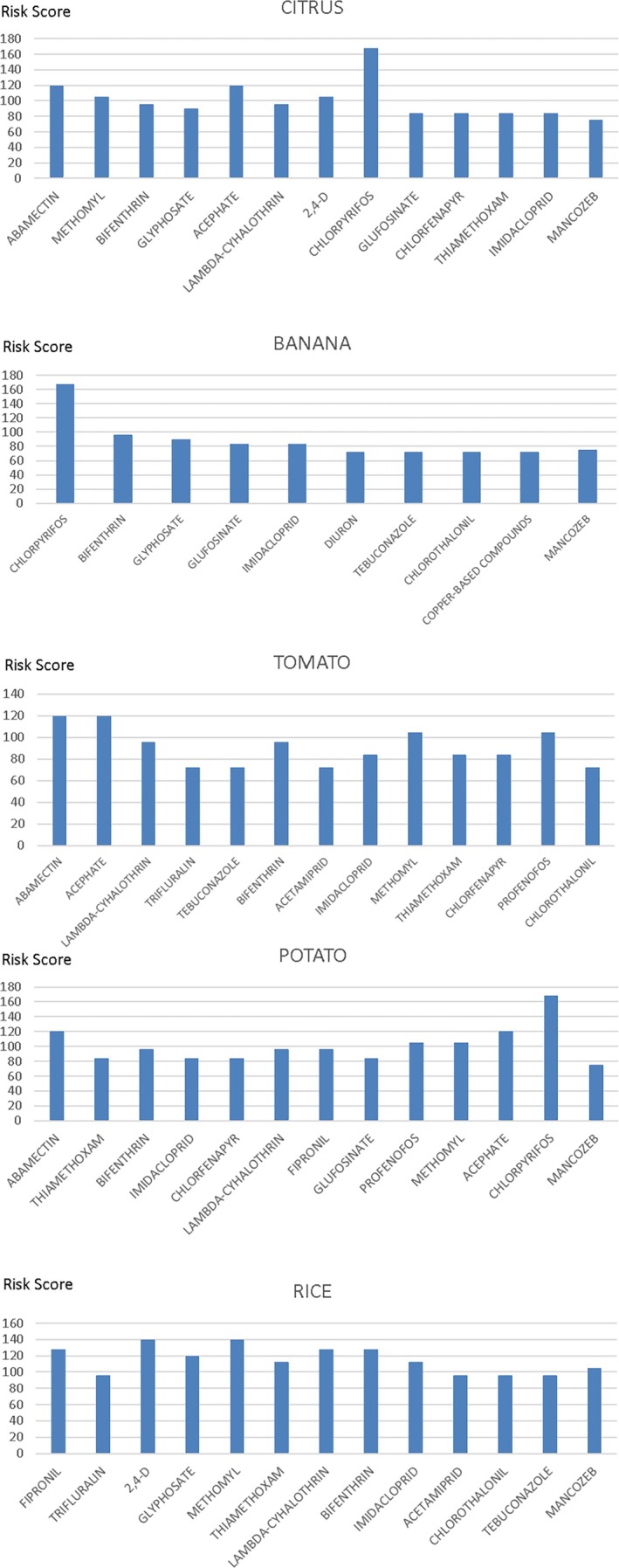
The five most consumed crops and their respective highest‐scoring pesticides.

Figure 1 presents the 12 pesticides that scored the highest and their respective associated crops. The five most consumed crops and their respective pesticides that reached the five highest risk scores are also presented (Fig. 2).

Different compounds presenting potential exposure risks, registered for application in the same crop, were observed in crops such as rice, citrus, potatoes, tomatoes, and bananas. This indicates the possibility of the presence of different residues in the same product. Chlorpyrifos presented the greatest potential risk for banana, citrus, and potato crops, followed by abamectin, acephate (for potato and citrus), and bifenthrin (for banana). This contrasts with a study carried out in Taiwan, which identified dithiocarbamates, endosulfan, and carbofuran as the greatest potential risk compounds to the health of the Taiwanese population.^41^ Endosulfan and carbofuran, however, are not registered in Brazil.^29^ Furthermore, the possibility that a specific substance may be present in different crops is also noteworthy, leading to exposure to the same pesticide from different sources.

In the PARA context, the selection of active ingredients to be investigated was carried out to meet the program's needs, considering the available analytical capacity and the rationalization of public resources. To this end, the following were evaluated: PARA's history of residue incidence, PNCRC results, data on pesticide marketing, and data from international programs.^33^ Data on toxicity and food consumption were apparently not considered for compound selection.

The PARA report for the 2017–2022 multi‐year plan presented the results of 5068 analyzed samples from 25 plant‐based foods, including zucchini, oats, bananas, onions, kale, oranges, apples, papaya, corn, cucumbers, pears, soybeans, grapes, peanuts, potatoes, broccoli, ground coffee, beans, cassava flour, passion fruit, strawberries, peppers, okra, cabbage, and wheat flour.^33^ Among the five most consumed foods depicted in Fig. 2, rice and tomatoes were not included in the 2018–2019 and 2022 cycles. Several methods can be used to identify potential exposure risks and depending on the purpose of the study and data availability, either quantitative, qualitative, or semi‐quantitative methods can be applied. Van der Fels‐Klerx *et al*. conducted a critical review of risk classification methods concerning chemical substances associated with food safety and food hazards for human healty.^28^ The scoring method uses semi‐quantitative scores for exposure and for human‐hazard health effects. When applying a scoring method, both exposure and severity endpoints are considered. However, the choice of exposure and effect endpoints can differ across studies because there is no scientific consensus on which endpoints should be included or on the criteria for their classification. As a result, the selection of appropriate endpoints for a given study is a critical step in ranking risks when employing a scoring method. Subsequently, suitable data sources, i.e. published literature, existing datasets, and/or expert judgment, must be identified to assign exposure and effect scores. This approach enables the integration of stakeholder perspectives in the scoring process. The relative importance attributed by each stakeholder to the model variables is captured through their assigned weights and, to ensure transparency, the basis for assigning weights should be clearly described and adequately documented. Thus, when applying a risk scoring method based on severity and probability factors, data concerning pesticides are classified on a scale ranging from low to high exposure risk, in which the scoring criteria can be adjusted or redefined according to each study.^28^ Risk classification studies are an important structured tool to enhance the residue detection process and are employed by several international agencies because they target combinations of foods and chemicals with the highest risk of exposure. However, these studies are based on calculation models and must be validated by comparison with usual approaches to assess increased detection.^28^, ^41^

The risk classification method presented herein was adapted from a method developed by the UK Veterinary Residues Committee (VRC), often used as a reference for ranking several chemical hazards because of its comprehensive semiquantitative risk matrix.^43^, ^44^, ^45^, ^46^, ^47^ The VRC classification matrix combines the nature of the hazard and potency (ADI), which is then multiplied by the sum of indicative exposure factors and the detectable residue evidence factor score.^44^, ^45^ The difference in the approach applied here was due to necessary adaptations based on available data.

Some inherent uncertainties in the applied data comprise limitations for risk classification methods, mainly linked to limited available data regarding residue occurrence and food consumption, the various publishing forms of monitoring programs results, and the lack of guidance values (in this case the ADI) for some compounds. In addition, the method considered only approved substances with the assumption that they are correctly used in crops authorized for use. Classification model caveats require continuous evaluations, adapting to the emergence of new and more robust data, to constantly improve risk prioritization tools.^43^

The classification result identified the compounds presenting the greatest risk of exposure, which should be prioritized in Brazilian monitoring programs.^33^ In this context, the introduction of lower‐priority substances into multi‐residue methods, commonly applied, and the development of specific methods for compounds presenting the greatest potential risk can be evaluated. It is also important to emphasize that substance prioritization should not exclude regulatory issues, misuse information, and relevant experiences from other countries.^43^

### 3.2 Survey of national food consumption data considering potential pesticide exposure food sources

The 2017–2018 POF was carried out based on two 24‐h recall surveys, conducted on non‐consecutive days chosen over the course of a week, to collect personal food consumption data. These surveys were applied in all Brazilian states and the Federal District. National food consumption was estimated considering the entire population, broken down by categories including sex, age groups (10–19 years old, 20–59 years old, 60 or older), monthly income level per family, housing contexts in urban or rural areas, and geographic division into Brazilian macro‐regions (North, Northeast, Southeast, South, Central‐West).^38^

Fresh foods are harvested directly from nature, whether from plants or animals (such as leaves, fruits, eggs, and milk) and are purchased for consumption without undergoing any modification. The selection of fresh vegetables covers specific categories, including fruits, vegetables, greens, roots, and tubers. Minimally processed foods undergo minimal procedures, such as the removal of inedible or unwanted parts, drying, dehydration, crushing or grinding, fractionation, roasting, cooking with water, pasteurization, chilling or freezing, careful packaging, vacuum packaging, and fermentation without producing alcohol.^48^

Data on average *per capita* food consumption (g/day) defined for each Brazilian macroregion were selected to compose the sensitivity analysis. Only natural and minimally processed foods were included in the sensitivity analysis due to food processing effects. Consumption data from national food surveys are more accurate than *per capita* household food acquisition data. However, to increase the study universe of sensitivity to plant‐based foods, acquisition data were used to complement the study with foods not defined in individual surveys.^32^, ^38^ Foods were grouped by primary crop, when applicable, and consumption/acquisition added up within each group.

### 3.3 ADI assessment of the contributions of different foods to dietary pesticides exposure

Sensitivity analyses were performed to assess which food contributed the most to pesticide residue exposure. This was carried out by calculating the relative contribution to exposure of each food in relation to other foods authorized for each compound. This strategy indicates the cumulative exposure to residues for each foodstuff, enabling monitoring program prioritization and optimization.^20^, ^41^

In addition, the average exposure of each food in relation to its guideline values (ADI) was calculated for each chemical compound.^20^ A lifetime body weight (average weight throughout life) of 60 kg was considered for this calculation.^18^ Only substances with MRLs defined for authorized pesticide use crops and with available consumption/acquisition data from the POF were subjected to the sensitivity analysis, performed considering the national context and the five Brazilian macro‐regions.

Sensitivity analyses were performed on pesticides that scored ≥10 in the risk matrix and met prerequisites considering long‐term daily exposure. Only chronic exposures were considered, applying average consumption/acquisition data to estimate exposure, therefore the ADI was used as a reference value for relative contribution calculations.^18^

Sensitivity calculations were performed for 2547 chemical compound and food correlations for each consumption scenario, both nationally and for the five Brazilian macro‐regions.

Most foods with a relative contribution ≥10 were common to all Brazilian regions. However, some foods were not common in both the national results and across all macroregions, as follows: cabbage and broccoli were classified as relevant in the calculations employing national data from the Southeast, South, and Central‐West regions; the consumption of broccoli, açaí, and coconut was noteworthy in the North region; kale was significant for residue exposure in the Southeast region; papaya in the South region; and lettuce in both the South and Southeast regions.

It is important to mention that mate tea, whose average consumption is high in the South region, was not subjected to a sensitivity assessment. Azadirachtin, an agrochemical used in mate cultivation, does not have an established MRL, which is required for assessing relative contributions. Its risk classification score was 12.

Many crops were not subjected to relative contribution assessments and average exposure calculations in relation to ADI values due to lack of consumption data. This is a study limitation and generates result uncertainties.

In one study, Jardim *et al*. assessed the acute cumulative dietary risk of organophosphate, carbamate, and pyrethroid insecticides for the Brazilian population. The authors indicate that dietary exposure assessment uncertainties are mainly due to a lack of accurate residue concentration and/or food consumption data, and that regional and seasonal differences must be considered.^49^ Another study estimated acute and chronic cumulative exposures to triazole and dithiocarbamate residues using a probabilistic approach, highlighting insufficient knowledge regarding exposure scenarios (concentration and/or consumption) in Brazil. The authors highlighted this as the study's main uncertainty factor.^22^, ^50^

**Figure 3:**
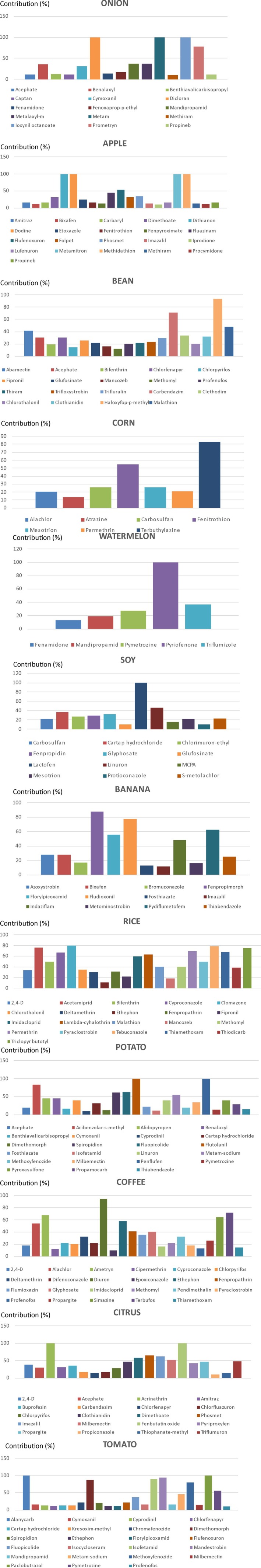
Sensitivity analysis results for the most critical pesticides.

Supporting Information, Table S2 depicts the foods with relative contributions ≥10 and their respective pesticides, along with the results related to national consumption. This includes some vegetables from the results obtained using regional consumption data. Figure 3 depicts the pesticides presenting the greatest potential risks associated with crops. Individual graphs were generated considering both the Brazilian consumption scenario and the crop results concerning regional consumption.

The average exposure calculation for each pesticide and their respective authorized crops ranged from 0% to 100% of their respective ADI, with some exceptions. Exposures to methyl bromide residues due to the consumption of citrus, coffee, watermelon, and apple ranged from 546% to 1418%, 1117% to 12 991%, 123% to 207%, and 103% of ADI values, respectively, considering both Brazilian and macro‐regional consumption. In addition, an average exposure of 124% of the ADI value was observed for zeta‐cypermethrin residues from rice consumption in the Central‐West region, and a variation from 311% to 469% of the ADI was detected for fluasifop‐*p*‐butyl residues due to bean consumption. These results were influenced mainly by the use of MRL values in average pesticide exposure calculations through the consumption of individual foods in relation to the ADI. Relatively low contributions when compared to other foods may, in themselves, result in residue concentrations above the ADI when food residue concentrations are considered. Highly consumed foods may not be the main source of exposure to a certain residue, an important variable when defining a model diet. Individual exposure assessments highlight the need for cumulative assessments, especially concerning associations where residue ingestion exceeds or approaches 100% of ADI values. Combined exposure assessments based on risk information allow for the selection of substances for grouping through a prioritization process, also allowing for the recognition of lower‐priority substances according to the relative contribution of each individual chemical to the total combined risk.^20^, ^51^, ^52^

When exposure to a certain residue exceeds ADI values, an individual's safety is not guaranteed regarding the adverse effects of that residue. The ADI is the estimate of the amount of pesticide residue in mg per kg of body weight that can be chronically ingested daily.^5^ In this study, average exposures were calculated considering a body weight of 60 kg. Smaller individuals and children may be even more exposed to these residues due to relative consumption rates per body weight, with children being particularly vulnerable.^53^

The establishment or confirmation of MRL values in Brazil results from pesticide residue studies. Studies on residues in plant‐based products are conducted on a specific pesticide applied to a specific crop for the purpose of registering with the competent federal authority. These studies include both field and laboratory phases. Field studies, conducted according to good laboratory practice (GLP) principles, are performed by simulating the correct use of the pesticide by farmers and are applied directly to a certain crop. The total amount applied must be in accordance with agronomic efficacy tests and instructions provided in the product's leaflet. For the establishment of an MRL value, tests are conducted to obtain a dissipation curve with the maximum recommended dose, which allows for the verification of the dissipation of the applied pesticide and related residues in the food over a certain period of time.^7^ Within the Codex scope, MRLs for pesticide residues are also derived from estimations considering toxicological evaluations of the pesticide and its residues and reviews concerning residue data from supervised trials and uses. Codex MRLs account for the highest levels demonstrated in these supervised trials, which are considered effective pest control practices. The evaluation makes it possible to verify whether the risk from exposure applying the MRL value is acceptable or not when compared to guidance values. Both in Brazil, through Anvisa, and within the Codex scope, different residue intake estimates from food are used to compare the MRL values to guidance values, such as the ADI, to demonstrate that the applied products are safe for human consumption.^6^, ^11^ Concerning pesticide residue MRL comparisons with the ADI, Anvisa and the JMPR, within the Codex Alimentarius framework, both calculate dietary intakes through the TMDI and the international estimation daily intake, respectively. For the TMDI calculation, *per capita* daily food consumption values, MRL values, average population body weights, processing factor and conversion factor values are employed. Results from average pesticide exposure estimates through individual foods above the ADI values should signal the unsuitability of the recommended maximum level values for certain residues.^11^, ^22^ However, it is important to note that, in the present study, the calculation of average exposure estimates was performed using the TMDI method, but processing and conversion factor values were not considered. This indicates only relevant data to be considered for refining the exposure estimate calculation.

Given the importance of the number of possible combinations between foods and residual substances, evaluating all combinations would require extensive technical and human support, in addition to consistent and detailed data. Substance prioritization and sensitivity studies facilitate the targeting of cumulative exposure assessments, aiming at more relevant food risk associations.^20^

Sensitivity assessments allow for optimization regarding sampling designs for the evaluated matrices, aiming at increasing cumulative exposure assessment efficiency. Although exposure is influenced by high average daily consumption rates, ensuring crucial food coverage, this alone does not provide information on the relative matrix contribution to exposure. This information is even more reliable when relative contributions are calculated with real occurrence data.^20^ In the present study, calculations were performed applying MRL values, and concentration variations in each matrix were not evaluated. Highly consumed foods may contain lower residue concentrations than less consumed foods.

Calculating relative contributions clarifies how much each foodstuff contributes to exposure to various residues critical to human health. This information can also be used to define the number of samples of each foodstuff to be included in residue occurrence surveys.

### 3.4 Proposal of a model diet(s) that meets the consumption particularities of different Brazilian regions

The consumption of certain foods is fundamentally linked with specific population exposure to certain residues. Consumption rates are therefore important in assessing human health risks.^54^

Some approaches can be used to define market baskets, many of which are based solely on high consumption information, that is, on dietary food contributions. Some methods use dietary food contributions as a criterion without considering sensitivity studies, while total diet studies represent entire diets, grouping foods and analyzing them as consumed, also without considering sensitivity studies. Another approach used to define GEMS/Food cluster diets is based on agricultural production information.^20^, ^55^, ^56^

The model diets proposed herein are based on the exposure principle, with the aim of optimizing substance prioritization in fresh food residue occurrence assessments. This approach allows for both dietary exposure assessments and MRL compliance but employs data on the relevance of each food to dietary exposure. Processing factors, if implemented, can optimize this approach to make it less conservative.

In cumulative exposure assessments, substances are defined and grouped based on scientific and regulatory criteria, which are formed by a preliminary group of common regulatory domains, and a risk‐oriented group that employs similar toxicological properties to group several chemical compounds. Risk‐based grouping requires a weight of evidence approach to evaluate and integrate available modes of action, adverse outcome pathways, phenomenological effects, and target organ/system toxicity evidence, among others.^22^, ^51^, ^57^ Therefore, considering the scarcity of toxicological data available for compound grouping, the approach applied herein was based on a grouping considering common regulatory domains, that is, only pesticides registered by MAPA.

A sensitivity analysis was performed to classify foods according to their relative residue exposure risk contributions, revealing foods that present greater dietary exposure risks.^54^ The model diets were thus defined by employing foods reaching ≥10% relative contribution values in the sensitivity study, according to Sieke.^20^

The foods reaching relative exposure contributions ≥10% of the total dietary intake of pesticide residues from a given chemical substance are presented in Table 2. The foods in bold are represented by vegetables that are not common to all Brazilian regions.

**Table 2:** Foods contributing ≥10% of the total dietary intake of pesticide residues from a given chemical substance

National	North	Northeast	Southeast	South	Central‐West
Rice	**Açaí**	Rice	**Lettuce**	**Lettuce**	Rice
Banana	Rice	Banana	Rice	Rice	Banana
Potato	Banana	Potato	Banana	Banana	Potato
**Broccoli**	Potato	Coffee	Potato	Potato	**Broccoli**
Coffee	**Broccoli**	Cana‐de‐açucar	**Broccoli**	**Broccoli**	Coffee
Sugarcane	Coffee	Onion	Coffee	Coffee	Sugarcane
Onion	Sugarcane	Citrus	Sugarcane	Sugarcane	Onion
Citrus	Onion	Bean	Onion	Onion	Citrus
Bean	Citrus	Apple	Citrus	Citrus	Bean
Apple	**Coconut**	Cassava	**Kale**	Bean	Apple
Cassava	Bean	Watermelon	Bean	Apple	Cassava
Watermelon	Apple	Corn	Apple	**Papaya**	Watermelon
Corn	Cassava	Soybean	Cassava	Cassava	Corn
**Cabbage**	Watermelon	Tomato	Watermelon	Watermelon	**Cabbage**
Soybean	Corn	Wheat	Corn	Corn	Soybean
Tomato	Soybean	–	**Cabbage**	**Cabbage**	Tomato
Wheat	Tomato	–	Soybean	Soybean	Wheat
–	Wheat	–	Tomato	Tomato	–
–	–	–	Wheat	Wheat	–

Foods in bold are not common to all Brazilian regions.

Considering that only minimal differences between the food groups indicated in the sensitivity study in the Brazilian and macroregion scenarios were observed, it is reasonable to adopt a single model diet to assess cumulative exposure for the following foods: açaí, lettuce, rice, banana, potato, broccoli, coffee, sugar cane, onion, citrus, coconut, kale, beans, apple, cassava, watermelon, corn, cabbage, soybeans, tomato, and wheat, correlated with available POF consumption data most representative of the Brazilian population (Table 3).

**Table 3:** Model diet for national use and respective national and regional consumption/acquisition data

Model diet	Consumption data/average *per capita* purchase (g day^−1^)					
	North	Northeast	South	Southeast	Central‐ West	National
Açaí	45.4	1.6	1.1	0.4	2.6	10.2
Lettuce	0.4	0.4	3.0	6.5	2.2	2.5
Rice	130.2	138.0	128.6	104.1	175.8	135.3
Banana	11.3	17.1	16.5	17.8	14.5	15.4
Potato	2.2	4.2	15.0	15.5	9.2	9.2
Broccoli	0.05	0.08	0.6	0.5	0.3	0.4
Coffee	135.3	151.9	173.7	184.6	134.0	155.9
Sugarcane (sugar)	28.1	32.2	30.1	28.4	26.6	29.6
Onion	2.3	9.7	9.9	7.7	8.3	8.5
Citrus (orange, tangerine, and lime)	11.9	10.9	28.3	15.6	15.1	16.5
Coconut	26.0	1.0	0.8	0.5	0.7	3.0
Kale	0.3	0.1	2.1	1.0	0.8	0.9
Bean	105.4	125.9	163.0	112.5	179.9	137.3
Apple	5.3	8.1	9.6	12.4	8.8	8.8
Papaya	3.5	6.3	6.0	7.9	5.9	5.9
Cassava (cassava and cassava flour)	43.0	28.0	6.2	15.3	14.5	21.4
Watermelon	4.3	6.2	2.4	3.7	5.1	4.3
Corn	6.8	37.6	7.9	13.0	8.3	14.7
Cabbage	0.1	0.2	1.4	3.6	1.9	1.4
Soybean (oil)	11.3	9.5	14.0	13.6	16.3	12.5
Tomato	1.2	1.7	4.6	8.3	6.3	4.4
Wheat(flour)	3.4	2.2	20.5	4.5	4.7	6.1

Source: IBGE32,38.

The chronic exposure assessment currently performed by JMPR is calculated according to the model diet established by the GEMS/Food cluster diets. To define model cluster diets, it is necessary to understand population food choices. However, food availability, geographic location (seasonality), and sociocultural environment directly influence dietary patterns. As an example, a study conducted in Tanzania found that low rainfall during dry and cold conditions is associated with food insecurity because access to food can be hindered due to reduced agricultural production and increased food prices. This can be exacerbated in families with a limited number of members of working age, especially in agriculture‐dependent populations. Another study evaluated the relationship between children's dietary patterns and family socioeconomic status, and concluded that children with a lower socioeconomic status consume fewer vitamin D food sources and more foods rich in fat and sugar.^15^, ^58^, ^59^ Therefore, the data to define cluster model diets are processed using two combined statistical methods to identify groups of target countries with similar dietary behaviors.^15^

The description of specific diets for each location is based on the WHO approach, which relies on the analysis of *per capita* supply data available from the FAO Food Balance Sheets. Supplies are the result of the total amount of produced food plus the total imported amount, adjusted for any stock variation, such as losses due to storage, transport, or other factors, during the selected period. In addition, exported amounts, those fed to livestock, used for seeds, or lost during storage and transport, and those available for human consumption are also considered. Thus, for a given food, *per capita* availability was calculated by dividing the difference between the supplied amounts and those used by the total population.^15^, ^60^ The 17 GEMS diets consist of national dietary patterns grouped based on similar consumption patterns, in turn based on the combination of several consumption systems. In this case, not all foods defined in a specific diet of a grouping are consumed by all individual countries within that grouping. However, in the absence of individual food consumption surveys, grouping makes it feasible for developing countries to implement better regional food safety measures and conduct baseline dietary exposure assessments regarding different food hazards.^60^

Another approach used to define a model diet (or market basket) comprises a total diet study (TDS). This can be used as a public health tool because it is possible to determine the chemical dietary exposure of a certain population. The fundamental principles of a TDS are to represent the entire diet and group foods, and analyze foods as consumed. In a TDS, the main foods are analyzed, prepared as consumed, and separated into food groups. The TDS steps include the selection of foods based on consumption data, their preparation as consumed, and the subsequent grouping of related foods prior to the analysis. Grouping is a critical step because it results in a single food sample for analysis, combining several individual food items. In addition, clustering influences both the accuracy and the cost of a TDS. It can be carried out through an individual dietary approach, grouping different forms of the same food, or through a mixed dietary approach using several different foods from the same food group combined into a single sample. A study aimed at assessing the occurrence and intake of organophosphate esters through the consumption of animal‐derived foods in China used a TDS conducted between 2016 and 2019 as a method for sampling and dietary intake assessment. A total of 14 organophosphate esters were detected in 96 food group composites referring to four categories of animal‐derived foods. The first harmonized total diet study in Portugal describes in detail the planning of the TDS, the collection of samples and their preparation. Lim *et al*. carried out a total diet study in Singapore covering the years 2021–2023. The paper provides an overview of the study design and methodological choices for conducting the TDS, describing the selection of chemical hazards and foods, as well as considerations regarding sampling and food preparation.^55^, ^61^, ^62^, ^63^ Consumption data are obtained from food consumption surveys of a certain population's diet. The main disadvantage is that this involves analyzing several foods grouped together. Mixing foods can cause a dilution effect, which occurs when a highly contaminated food is combined with several minimally contaminated foods. The resulting samples of grouped foods may not present measurable concentration values.^55^

Benbrook and Davis developed a system called the Dietary Risk Index (DRI) to facilitate pesticide residue data analyses from the US and UK, as well as tracking trends and chronic risk distributions. The DRI value is the result of pesticide intake through the consumption of a single serving of food containing the residue divided by the respective ADI established by the United States Environmental Protection Agency. The calculation is based on the annual average levels of occurrence of a given residue in monitoring programs (the US Pesticide Data Program and the UK Food Standards Agency) and on individual residue levels in food samples. In addition, DRI values can be calculated from the perspective of a specific food containing multiple chemicals or a specific substance in different food samples. The DRI system is limited to data on actual and measured residue occurrence from the UK and US monitoring programs. Therefore, in addition to considering consumption and occurrence data specific to these regions, cumulative exposures to multiple substances in all foods in which a pesticide is registered for use are not fully considered in the DRI calculation.^64^, ^65^

### 3.5 Exposure assessments employing the GEMS/food cluster diets group 5 model diet and the model diet proposed in this study

An approach considering grouping from a common regulatory domain was applied. A comparison was carried out regarding chronic pesticide exposure calculations between the proposed diet and the GEMS/Food diet consumption data.^17^ The MRL values and a body weight of 60 kg were used for all calculations. Additionally, for the proposed diet, average food consumption *per capita* (g day^−1^) values for the Brazilian population according to the POF 2017–2018 were used. The risk characterization was performed by calculating the HI.

According to Anvisa, the assessment of chronic dietary exposure in Brazil should be conducted using equations to estimate the TMDI. When monitoring data are used, the concentrations of sample pesticide residues are considered instead of median residues from field studies or MRL values. The use of monitoring data allows for a real understanding of the risks arising from dietary residue exposures. Average values are adopted for consumption and body weight data.^11^, ^33^

A total of 27 pesticides posing potential health risks were randomly selected to undergo a TMDI calculation. No processing factors were considered. Additionally, a risk characterization was conducted by calculating HI. The results considering the consumption data of the proposed model diet and the GEMS/Food cluster diet were compared.

The TMDI results for the proposed diet were 0–0.13 mg and the HI was 138.7 (Table 4). In contrast, the GEMS/Food group 5 diet TMDI results ranged from 0 to 0.017 mg and the HI was 21.1 (Table 5). Both results would represent a potential cumulative health risk if they were real cases (HI > 1), in contrast to an individual assessment. Additionally, the results of both approaches were significantly different.

**Table 4:** Results of the TMDI and hazard index calculations for the proposed diet

Active ingredient	Crop	Proposed dietary exposure (mg kg^−1^)	TMDI (mg kg^−1^ bw)	HQ
2,4‐D	Rice	0.029	0.0013	0.134
	Coffee	0.016		
	Corn	0.003		
	Citrus	0.033		
Acephate	Potato	0.002	0.0002	0.145
	Onion	0.001		
	Corn	0.000		
	Citrus	0.003		
	Bean	0.003		
Acetamiprid	Rice	0.432	0.0072	0.301
	Corn	0.001		
	Broccoli	0.000		
Alpha‐cypermethrin	Rice	0.288	0.0056	0.280
	Corn	0.001		
	Coffee	0.047		
Benfuracarb	Sugarcane	0.003	0.0000	0.328
Bifenthrin	Rice	0.216	0.0050	0.252
	Corn	0.000		
	Bean	0.086		
Bixafen	Banana	0.031	0.0018	0.886
	Coffee	0.047		
	Bean	0.015		
	Apple	0.013		
Methyl bromide	Coffee	7.795	0.1299	129.917
Clethodim	Coffee	0.078	0.0040	0.399
	Corn	0.007		
	Bean	0.154		
Chlorfenapyr	Citrus	0.008	0.0005	0.018
	Corn	0.001		
	Bean	0.017		
	Tomato	0.007		
Chlorfluazuron	Citrus	0.002	0.0001	0.024
	Corn	0.000		
	Bean	0.002		
	Cabbage	0.001		
	Tomato	0.002		
Chlorothalonil	Rice	0.576	0.0153	0.510
	Corn	0.000		
	Bean	0.342		
Deltamethrin	Rice	0.144	0.0062	0.624
	Coffee	0.156		
	Corn	0.015		
	Bean	0.060		
Dimethoate	Citrus	0.033	0.0008	0.423
	Apple	0.018		
Diuron	Corn	0.001	0.0000	0.002
	Coffee	0.156		
Fenitrothion	Apple	0.004	0.0004	0.084
	Corn	0.015		
	Wheat	0.006		
Fipronil	Rice	0.001	0.0001	0.349
	Corn	0.000		
	Sugarcane	0.001		
	Bean	0.002		
Glyphosate	Coffee	0.156	0.0049	0.010
	Corn	0.015		
	Soybean	0.126		
Haloxyfop‐*p*‐methyl	Bean	0.009	0.0001	0.475
Metam	Onion	0.013	0.0002	0.213
Metam‐sodium	Potato	0.011	0.0003	0.327
	Tomato	0.009		
Methomyl	Rice	0.029	0.0009	0.045
	Coffee	0.016		
	Corn	0.001		
	Bean	0.009		
Pirimiphos‐methyl	Corn	0.074	0.0132	0.441
	Rice	0.721		
Propargite	Coffee	0.047	0.0022	0.216
	Citrus	0.083		
Propineb	Onion	0.013	0.0062	1.242
	Bean	0.342		
	Apple	0.018		
Protioconazole	Bean	0.010	0.0002	0.218
	Corn	0.000		
	Soybean	0.003		
Terbufos	Coffee	0.008	0.0002	0.853
	Corn	0.001		
	Bean	0.002		
**Hazard index calculation: 138.7**				

2,4‐D, 2,4‐dichlorophenoxy)acetic acid; bw, body weight; HQ, hazard quotient; TMDI, theoretical maximum daily intake.

**Table 5:** Results of the TMDI and hazard index calculations for the GEMS/Food diet

Active ingredient	Crop	Exposure GEMS/Food (mg kg^−1^)	TMDI (mg kg^−1^ bw)	HQ
2,4‐D	Rice	0.072	0.0035	0.352
	Coffee	0.000		
	Corn	0.072		
	Citrus	0.067		
Acephate	Potato	0.013	0.0005	0.433
	Onion	0.003		
	Corn	0.007		
	Citrus	0.007		
	Bean	0.001		
Acetamiprid	Rice	1.075	0.0182	0.760
	Corn	0.018		
	Broccoli	0.002		
Alpha‐cypermethrin	Rice	0.717	0.0123	0.613
	Corn	0.018		
	Coffee	0.001		
Benfuracarb	Sugarcane	0.009	0.000	0.958
Bifenthrin	Rice	0.538	0.0094	0.468
	Corn	0.007		
	Bean	0.017		
Bixafen	Banana	0.186	0.0034	1.676
	Coffee	0.001		
	Bean	0.003		
	Apple	0.011		
Methyl bromide	Coffee	0.092	0.0015	1.537
Clethodim	Coffee	0.001	0.0035	0.350
	Corn	0.179		
	Bean	0.030		
Chlorfenapyr	Citrus	0.017	0.0018	0.060
	Corn	0.018		
	Bean	0.003		
	Tomato	0.069		
Chlorfluazuron	Citrus	0.003	0.0008	0.160
	Corn	0.004		
	Bean	0.000		
	Cabbage	0.018		
	Tomato	0.023		
Chlorothalonil	Rice	1.434	0.0251	0.835
	Corn	0.004		
	Bean	0.066		
Deltamethrin	Rice	0.358	0.0122	1.217
	Coffee	0.002		
	Corn	0.358		
	Bean	0.012		
Dimethoate	Citrus	0.067	0.0014	0.688
	Apple	0.015		
Diuron	Corn	0.018	0.0003	0.043
	Coffee	0.002		
Fenitrothion	Apple	0.004	0.0120	2.402
	Corn	0.358		
	Wheat	0.358		
Fipronil	Rice	0.004	0.0002	0.840
	Corn	0.004		
	Sugarcane	0.003		
	Bean	0.000		
Glyphosate	Coffee	0.002	0.0063	0.013
	Corn	0.358		
	Soybean	0.016		
Haloxyfop‐*p*‐methyl	Bean	0.002	0.0000	0.092
Metam	Onion	0.032	0.0005	0.527
Metam‐sodium	Potato	0.067	0.0027	2.656
	Tomato	0.092		
Methomyl	Rice	0.072	0.0018	0.091
	Coffee	0.000		
	Corn	0.036		
	Bean	0.002		
Pirimiphos‐methyl	Corn	1.792	0.0597	1.991
	Rice	1.792		
Propargite	Coffee	0.001	0.0028	0.282
	Citrus	0.168		
Propineb	Onion	0.032	0.0019	0.377
	Bean	0.066		
	Apple	0.015		
Protioconazole	Bean	0.002	0.0002	0.158
	Corn	0.007		
	Soybean	0.000		
Terbufos	Coffee	0.000	0.0003	1.529
	Corn	0.018		
	Bean	0.000		
**Hazard index calculation: 21.1**				

2,4‐D, 2,4‐dichlorophenoxy)acetic acid; bw, body weight; GEMS, Global Environment Monitoring System; HQ, hazard quotient; TMDI, theoretical maximum daily intake.

The GEMS/Food program collaborates with over 30 esteemed national centers and institutions worldwide, monitoring the levels and trends of chemical substances in food and assessing their contribution to dietary exposure by compiling data from its collaborators. The program's diets represent the average *per capita* food consumption for 17 country groupings, each consisting of two to 30 nations. The amount of food available for consumption within each group is divided by its total population. These 17 diets are appropriate for estimating the general population's chronic dietary exposure to pesticide residues in food, based on average consumption,^16^, ^17^, ^66^ therefore exposure assessment calculations based on international data should provide exposure estimates similar to or higher than estimates at the national level. In other words, if the estimated international chronic dietary exposure to a chemical substance does not exceed the corresponding health‐based guidance value, the exposure level will be acceptable at each national level where more refined data may be available.^15^ However, the results presented herein do not support this statement (HI of 21.1), even when exposure calculations are performed considering all authorized crops with a chemical risk score of ≥10 for the 27 selected pesticides (HI of 88.23).

The proposed model diet includes key foods for the Brazilian population, such as coffee, soy, and beans, with consumption levels that better reflect national dietary habits. In contrast, the GEMS/Food cluster diet represents them at very low consumption levels compared with actual Brazilian consumption. Consequently, the use of the GEMS/Food cluster diet may lead to pesticide exposure underestimations in cumulative dietary risk assessments under the Brazilian scenario. It is important to note that specific foods are not identified in the GEMS/Food diet, which instead relies on broad food categories. For example, all tropical fruits are considered as consumed by the population in the same amounts, which makes exposure assessments more challenging.^15^, ^17^, ^66^

The detection of different pesticide residues in dietary samples is relevant, especially concerning substances leading to the same adverse health effects. The latest PARA report, prepared by Anvisa, presented the number of detected pesticide residues, both at regular and irregular levels, in Brazilian samples. Two or more pesticide residues were detected simultaneously in 50.4% of the total samples analyzed, with 10 or more active substances found in 62 samples (2%). The report thus highlights the need to formulate guidelines to verify whether the occurrence of multiple residues contributes to the extrapolation of safe exposure reference values.^33^

In the case of dual active pharmaceutical ingredients, chronic exposure assessments must consider both their use as veterinary drugs and as agrochemicals. In this case, the calculations resulting from the respective exposures must be added in the TMDI calculation.^67^

The methodology employed in toxicity accumulation assessments is important in evaluating cumulative risk because it appraises combined exposures to different compounds expressed as functions of their toxicities. To refine cumulative exposure assessments and reduce uncertainties, it is essential to generate and provide concrete toxicological data to establish CAGs, comprising a group of substances that all present a common toxic characteristic to be evaluated, such as mechanism/mode of action, adverse effect pathway, phenomenological effect, and common adverse effects on target organs and systems, among others. Grouping methods are used to combine exposure toxicity to different substances within the same CAG and perform cumulative assessments. The HI, adjusted hazard index, reference point index, relative potency factors, or physiologically based toxicokinetic and toxicodynamic modeling calculations are employed. After composing the CAG, cumulative dietary residue exposure assessments regarding this group of substances are performed.^22^, ^52^, ^68^, ^69^

A hierarchy of approaches is noted when applying exposure assessments, depending on the available information and study objectives. At the lowest level, a more conservative approach is employed, relying on upper‐bound exposure estimates for each pesticide. At higher levels, actual exposure correlations are performed employing monitoring and modeling data, and exposure studies for specific scenarios. The level of result refinement and accuracy is proportional to the level of the approach and to the characteristics of the applied occurrence and consumption data, which range from 0 (point‐based and semi‐quantitative) to 1 (deterministic), 2 (semi‐probabilistic), and 3 (probabilistic).^22^, ^52^, ^57^

Risk characterization methodologies also vary according to cumulative exposure estimate levels, although all compare the sum of individual chemical exposures with reference values. At level 0, the HI calculation is commonly employed for risk characterizations, expressed as the sum of the hazard quotients calculated for each component of a cumulative exposure. The hazard quotient comprises the ratio between the estimated exposure to a chemical and its respective reference value (ADI and tolerable daily intake or the lowest available reference value). Typically, HI ≤1 results indicate that the combined risk is acceptable. When HI exceeds 1, potential concerns are noted and may indicate the need for more refined assessments. The HI calculation is also applied at level 1, using the respective reference values. In scenarios where the employed database presents a higher level of detail, it is possible to calculate the target organ toxicity dose using an HI approach. The internal dose HI can also be calculated when exposure is corrected for parameters such as absorption or body load and clearance, among others. At higher exposure assessment levels, available toxicokinetic data allow for more refined evaluations and risk metrics are treated in a more quantitative and probabilistic manner.^22^, ^57^, ^68^, ^70^

Projects for the assessment of toxicological data are being carried out, albeit gradually. Currently, CAGs for defined pesticides are still scarce. However, CAGs generated from the grouping of substances presenting the same mechanisms/modes of action and based on effects on a common target organ/system can be found.^71^

Considering the discussion above and the conservative exposure estimate results obtained herein, the calculations in a real case would indicate potential risks and the need to raise the assessment level. Refining assessments offers more robust foundations for decision‐making by managers. In the present study, a hypothetical scenario was assumed in which an individual was exposed to the estimated upper exposure limit of the 27 evaluated pesticides, with the aim of verifying the applicability of the proposed model diet compared to the international consumption alternative. In this sense, the use of the proposed model diet proved advantageous in representing the national consumption scenario.

A previously published study concluded that the main challenges for cumulative pesticide exposure assessments in Brazil comprise the following: lack of consistent occurrence and consumption data, absence of a legal basis with specific guidelines and requirements for this type of assessment, lack of a specific methodology adapted to the national scenario, and the need to prioritize chemical substances and foods in residue monitoring and surveillance programs. According to the authors, consumption and occurrence data must reflect the significant regional diversity of Brazil. Given the relevance of the topic to both national and global public health, regulatory agencies should prioritize the development of standards that require combined exposure assessments to multiple substances in food while also providing clear guidelines on how to conduct these assessments.^22^ Therefore, the findings reported herein, which involve pesticide prioritization and the proposal of a model diet capable of considering consumption particularities in different Brazilian regions, contribute to addressing the challenges associated with cumulative pesticide exposure assessments in the country.

## 4 CONCLUSION

A classification assessment of pesticides was conducted by applying the scoring method approach to rank chemical hazard‐crop combinations to assess cumulative dietary exposure. A sensitivity analysis was performed to identify which foodstuffs contribute most as sources of residue exposure. A model diet that approximates Brazilian eating habits, considering the exposure principle, was proposed to optimize substance prioritization in residue occurrence assessments for natural foods. A practical simulation with pesticides demonstrated that the use of the GEMS/Food group diet, when applied specifically for Brazil, may underestimate pesticide exposure when performing a chronic cumulative exposure assessment.

## CONFLICT OF INTEREST

The authors have declared no conflict of interests.

## AUTHOR CONTRIBUTIONS

**Bianca Figueiredo de Mendonça Pereira:** Conceptualization, formal analysis, investigation, data curation, writing – original draft, visualization. **Bernardete Ferraz Spisso:** Conceptualization, writing – review and editing, visualization, supervision, project administration.

## Supporting information


**Table S1.** Pesticide risk matrix.

**Table S2.** Foodstuffs with relative contributions ≥10% in the sensitivity analysis and their respective pesticides.

## Data Availability

The data that supports the findings of this study are available in the supplementary material of this article.
