# Supplementary material for: Pesticide prioritization based on risk and model diet proposals for assessing cumulative exposure to pesticide residues in the Brazilian population through food consumption
Source: Pest Manag Sci. 2026 Jan 13;82(4):3853–73. doi: 10.1002/ps.70507 (PMC12976163; doi:10.1002/ps.70507)
Supplement: Supplementary file 2 — Table S2. Foodstuffs with relative contributions ≥10% in the sensitivity analysis and their respective pesticides. [file PS-82-3853-s002.docx]

**Supplemntary Information for: "Pesticide prioritization based on risk and model diet proposals for assessing cumulative exposure to pesticide residues in the Brazilian population through food consumption"**

**Bianca Figueiredo de Mendonça Pereira; Bernardete Ferraz Spisso***

***Corresponding author: Bernardete Ferraz Spisso, Ph.D. (bernardete.spisso@fiocruz.br)**

**Table S2.** Foodstuffs with relative contributions ≥ 10% in the sensitivity analysis and their respective pesticides.

| **Crop** | **Active Ingredient** | **Relative Contribution of Food to Pesticide Exposure Combined (%)** | | **Average Pesticide Exposure Through Individual Foods in Relation to the ADI (%)** | **Final Score (Risk Matrix)** |
| --- | --- | --- | --- | --- | --- |
| Açaí | Diafenthiuron | 22.12 | 25.22 | | 12 |
| Lettuce | Azoxystrobin | 12.77 | 10.8 | | 12 |
| Lettuce | Fluazinam | 10.43 | 2.2 | | 15 |
| Lettuce | Fluxapyroxad | 19.72 | 3.8 | | 20 |
| Lettuce | Methiram | 12.11 | 1.1 | | 15 |
| Lettuce | Pyraclostrobin | 13.58 | 2.7 | | 16 |
| Lettuce | Procymidone | 18.49 | 0.5 | | 15 |
| Lettuce | Thiram | 16.77 | 1.4 | | 28 |
| Rice | 2,4-D | 33.54 | 4.8 | | 140 |
| Rice | Acetamiprid | 75.82 | 30.0 | | 96 |
| Rice | Alpha-cypermethrin | 72.96 | 24.0 | | 14 |
| Rice | Azimsulfuron | 100.00 | 0.2 | | 16 |
| Rice | Azoxystrobin | 14.00 | 9.6 | | 12 |
| Rice | Bentazone | 85.50 | 0.2 | | 48 |
| Rice | Benzovindiflupyr | 71.30 | 3.8 | | 48 |
| Rice | Beta-cyfluthrin | 52.78 | 4.8 | | 32 |
| Rice | Beta-cypermethrin | 76.14 | 48.0 | | 20 |
| Rice | Bifenthrin | 49.70 | 18.0 | | 128 |
| Rice | Bispyribac | 70.91 | 1.2 | | 20 |
| Rice | Carboxin | 40.59 | 0.5 | | 20 |
| Rice | Carfentrazone-ethyl | 73.42 | 0.2 | | 16 |
| Rice | Kasugamycin | 89.57 | 0.1 | | 16 |
| Rice | Cyhalofop-butyl | 100.00 | 0.8 | | 16 |
| Rice | Cyantraniliprole | 14.03 | 0.2 | | 16 |
| Rice | Cyclosulfamuron | 100.00 | 1.6 | | 16 |
| Rice | Cyfluthrin | 34.51 | 1.2 | | 36 |
| Rice | Cipermethrin | 77.34 | 9.6 | | 14 |
| Rice | Cyproconazole | 66.57 | 24.0 | | 84 |
| Rice | Clomazone | 79.67 | 0.6 | | 72 |
| Rice | Chlorantraniliprole | 67.22 | 0.2 | | 24 |
| Rice | Chlorothalonil | 34.49 | 32.0 | | 96 |
| Rice | Kresoxim-methyl | 86.70 | 0.0 | | 12 |
| Rice | Deltamethrin | 30.04 | 24.0 | | 64 |
| Rice | Difenoconazole | 39.93 | 0.4 | | 60 |
| Rice | Diflubenzuron | 15.84 | 1.2 | | 60 |
| Rice | Dinotefuran | 81.74 | 16.4 | | 24 |
| Rice | Epoxiconazole | 80.29 | 72.1 | | 60 |
| Rice | Esfenvalerate | 85.39 | 12.0 | | 28 |
| Rice | Ethephon | 10.66 | 1.0 | | 80 |
| Rice | Etofenprox | 76.05 | 24.0 | | 20 |
| Rice | Ethoxysulfuron | 100.00 | 0.3 | | 16 |
| Rice | Fenoxaprop-p-ethyl | 35.23 | 4.8 | | 20 |
| Rice | Fenpropathrin | 30.74 | 3.2 | | 64 |
| Rice | Fipronil | 21.75 | 12.0 | | 128 |
| Rice | Flutriafol | 17.17 | 2.4 | | 24 |
| Rice | Fluxapyroxad | 42.63 | 7.2 | | 10 |
| Rice | Phthalide | 100.00 | 1.6 | | 16 |
| Rice | Maleic hydrazide | 100.00 | 8.0 | | 12 |
| Rice | Fentin | 59.52 | 48.0 | | 36 |
| Rice | Imazamox | 100.00 | 0.2 | | 12 |
| Rice | Imazapic | 100.00 | 0.0 | | 16 |
| Rice | Imazethapyr | 28.19 | 0.0 | | 24 |
| Rice | Imidacloprid | 59.38 | 9.6 | | 112 |
| Rice | Iodosulfuron-methylsodium | 100.00 | 0.1 | | 16 |
| Rice | Ipconazole | 93.05 | 0.2 | | 16 |
| Rice | Lambda-cyhalothrin | 62.70 | 4.8 | | 128 |
| Rice | Malathion | 40.65 | 6.4 | | 96 |
| Rice | Mancozeb | 18.60 | 6.4 | | 100 |
| Rice | Mefentrifluconazole | 94.23 | 10.3 | | 16 |
| Rice | Metalaxyl-m | 12.71 | 0.0 | | 24 |
| Rice | Metamifop | 100.00 | 0.9 | | 16 |
| Rice | Methomyl | 40.01 | 2.4 | | 140 |
| Rice | Metominostrobin | 53.64 | 8.8 | | 32 |
| Rice | Metsulfuron-methyl | 94.57 | 0.5 | | 16 |
| Rice | Myclobutanil | 76.52 | 4.0 | | 24 |
| Rice | Novaluron | 88.65 | 16.8 | | 16 |
| Rice | Orthosulfamuron | 100.00 | 0.1 | | 16 |
| Rice | Oxadiazon | 87.64 | 3.3 | | 20 |
| Rice | Oxyfluorfen | 39.25 | 4.0 | | 20 |
| Rice | Pendimethalin | 14.85 | 0.1 | | 14 |
| Rice | Penoxsulam | 100.00 | 0.0 | | 16 |
| Rice | Permethrin | 69.26 | 4.8 | | 64 |
| Rice | Picloram | 92.30 | 0.1 | | 36 |
| Rice | Picoxystrobin | 56.21 | 0.4 | | 10 |
| Rice | Pyraclostrobin | 49.12 | 9.0 | | 64 |
| Rice | Pyrazosulfuron-ethyl | 100.00 | 0.0 | | 16 |
| Rice | Pirimiphos-methyl | 84.16 | 40.0 | | 56 |
| Rice | Profoxydim | 100.00 | 2.4 | | 20 |
| Rice | Propanil | 100.00 | 24.0 | | 24 |
| Rice | Propaquizafop | 100.00 | 0.8 | | 16 |
| Rice | Propiconazole | 52.84 | 1.8 | | 12 |
| Rice | Quinclorac | 100.00 | 0.0 | | 16 |
| Rice | Quizalofop-p-ethyl | 72.37 | 8.0 | | 24 |
| Rice | Saflufenacil | 24.40 | 0.2 | | 20 |
| Rice | Tebuconazole | 79.06 | 48.0 | | 96 |
| Rice | Teflubenzuron | 59.53 | 2.4 | | 16 |
| Rice | Tetraconazole | 53.96 | 48.0 | | 28 |
| Rice | Thiamethoxam | 67.38 | 12.0 | | 112 |
| Rice | Thiodicarb | 38.56 | 0.8 | | 64 |
| Rice | Thiophanate-methyl | 12.71 | 1.5 | | 60 |
| Rice | Thiram | 48.44 | 3.8 | | 14 |
| Rice | Tricyclazole | 100.00 | 28.7 | | 48 |
| Rice | Triclopyr butotyl | 74.99 | 0.4 | | 72 |
| Rice | Trifloxystrobin | 28.90 | 2.4 | | 12 |
| Rice | Trifluralin | 24.74 | 0.5 | | 96 |
| Rice | Florpyrauxifen-benzyl | 100.00 | 0.0 | | 12 |
| Rice | Imazapyr | 100.00 | 0.0 | | 12 |
| Rice | Zeta-cypermethrin | 75.55 | 96.1 | | 64 |
| Banana | Azoxystrobin | 28.13 | 19.3 | | 12 |
| Banana | Bixafen | 27.97 | 25.7 | | 24 |
| Banana | Bromuconazole | 17.18 | 1.3 | | 18 |
| Banana | Fenpropimorph | 87.53 | 17.2 | | 10 |
| Banana | Florylpicoxamid | 56.00 | 0.3 | | 12 |
| Banana | Fludioxonil | 77.38 | 19.3 | | 12 |
| Banana | Fosthiazate | 12.68 | 1.3 | | 15 |
| Banana | Imazalil | 11.68 | 0.9 | | 42 |
| Banana | Indaziflam | 48.26 | 0.0 | | 12 |
| Banana | Metominostrobin | 16.42 | 2.7 | | 24 |
| Banana | Pydiflumetofem | 62.67 | 0.4 | | 12 |
| Banana | Thiabendazole | 25.46 | 1.5 | | 18 |
| Potato | Acephate | 19.32 | 3.0 | | 120 |
| Potato | Acibenzolar-s-methyl | 82.97 | 0.4 | | 12 |
| Potato | Afidopyropen | 44.82 | 0.0 | | 12 |
| Potato | Benalaxyl | 45.46 | 0.0 | | 15 |
| Potato | Benthiavalicarbisopropyl | 15.88 | 0.0 | | 15 |
| Potato | Cymoxanil | 39.66 | 0.2 | | 18 |
| Potato | Cyprodinil | 10.08 | 0.1 | | 12 |
| Potato | Cartap hydrochloride | 31.82 | 0.0 | | 12 |
| Potato | Dimethomorph | 12.76 | 0.0 | | 12 |
| Potato | Spiropidion | 61.13 | 0.2 | | 12 |
| Potato | Fluopicolide | 63.04 | 0.0 | | 12 |
| Potato | Flutolanil | 100.00 | 0.0 | | 12 |
| Potato | Fosthiazate | 22.11 | 2.2 | | 15 |
| Potato | Isofetamid | 10.86 | 0.0 | | 12 |
| Potato | Linuron | 39.30 | 6.0 | | 18 |
| Potato | Metam-sodium | 54.92 | 18.0 | | 24 |
| Potato | Methoxyfenozide | 19.59 | 0.0 | | 12 |
| Potato | Milbemectin | 33.93 | 0.0 | | 15 |
| Potato | Penflufen | 100.00 | 0.0 | | 12 |
| Potato | Pymetrozine | 13.40 | 0.1 | | 18 |
| Potato | Pyroxasulfone | 39.41 | 0.2 | | 12 |
| Potato | Propamocarb | 28.88 | 0.1 | | 12 |
| Potato | Thiabendazole | 14.80 | 0.9 | | 18 |
| Potato | Thifluzamide | 100.00 | 0.1 | | 12 |
| Potato | Ametoctradin | 70.90 | 0.0 | | 12 |
| Broccoli | Mandipropamid | 19.47 | 0.1 | | 16 |
| Coffee | 2,4-D | 18.14 | 2.6 | | 70 |
| Coffee | Acetochlor | 88.10 | 5.2 | | 12 |
| Coffee | Alachlor | 54.35 | 1.3 | | 32 |
| Coffee | Alpha-cypermethrin | 11.84 | 3.9 | | 14 |
| Coffee | Ametryn | 67.42 | 0.2 | | 36 |
| Coffee | Emamectin benzoate | 24.87 | 5.2 | | 21 |
| Coffee | Beta-cypermethrin | 12.36 | 7.8 | | 15 |
| Coffee | Bixafen | 42.37 | 39.0 | | 16 |
| Coffee | Boscalid | 25.74 | 0.3 | | 16 |
| Coffee | Methyl bromide | 92.55 | 12991.7 | | 15 |
| Coffee | Cadusafos | 89.32 | 43.3 | | 20 |
| Coffee | Cyclaniliprole | 53.58 | 0.2 | | 10 |
| Coffee | Cyflumetofen | 31.29 | 0.0 | | 15 |
| Coffee | Cyfluthrin | 18.67 | 0.6 | | 18 |
| Coffee | Cipermethrin | 12.55 | 1.6 | | 56 |
| Coffee | Cyproconazole | 21.61 | 7.8 | | 42 |
| Coffee | Clethodim | 17.09 | 13.0 | | 12 |
| Coffee | Chlorantraniliprole | 14.54 | 0.0 | | 12 |
| Coffee | Chlorimuron-ethyl | 66.28 | 0.0 | | 15 |
| Coffee | Chlorpyrifos | 20.24 | 3.9 | | 112 |
| Coffee | Deltamethrin | 32.49 | 26.0 | | 32 |
| Coffee | Diafenthiuron | 17.40 | 17.3 | | 12 |
| Coffee | Difenoconazole | 21.60 | 0.2 | | 30 |
| Coffee | Diuron | 93.98 | 37.1 | | 48 |
| Coffee | Epoxiconazole | 9.65 | 8.7 | | 30 |
| Coffee | Spinetoram | 12.03 | 0.3 | | 10 |
| Coffee | Spinosad | 79.66 | 2.6 | | 18 |
| Coffee | Spirodiclofen | 10.32 | 1.0 | | 12 |
| Coffee | Spiromesifen | 51.59 | 2.9 | | 16 |
| Coffee | Ethephon | 57.65 | 5.2 | | 40 |
| Coffee | Ethiprole | 83.70 | 5.2 | | 16 |
| Coffee | Etoxazole | 44.65 | 0.1 | | 10 |
| Coffee | Fenpyroximate | 24.40 | 1.3 | | 14 |
| Coffee | Fenpropathrin | 41.57 | 4.3 | | 32 |
| Coffee | Flazasulfuron | 56.75 | 0.6 | | 15 |
| Coffee | Fluensulfone | 65.57 | 5.2 | | 12 |
| Coffee | Flumioxazin | 35.52 | 0.6 | | 36 |
| Coffee | Fluopyram | 11.46 | 0.6 | | 15 |
| Coffee | Flupyradifurone | 55.28 | 13.0 | | 21 |
| Coffee | Fluxapyroxad | 15.37 | 2.6 | | 10 |
| Coffee | Fomesafen | 14.30 | 0.9 | | 24 |
| Coffee | Fosthiazate | 64.00 | 6.5 | | 10 |
| Coffee | Gamma-cyhalothrin | 38.99 | 13.0 | | 15 |
| Coffee | Glyphosate | 40.37 | 0.5 | | 60 |
| Coffee | Imidacloprid | 16.06 | 2.6 | | 56 |
| Coffee | Indoxacarb | 18.88 | 1.8 | | 14 |
| Coffee | Iprodione | 74.59 | 8.7 | | 12 |
| Coffee | Lufenuron | 22.51 | 0.6 | | 12 |
| Coffee | Metaflumizone | 24.53 | 0.9 | | 15 |
| Coffee | Metconazole | 71.41 | 1.1 | | 12 |
| Coffee | Methomyl | 21.64 | 1.3 | | 70 |
| Coffee | Metribuzim | 69.80 | 2.0 | | 28 |
| Coffee | Myclobutanil | 16.56 | 0.9 | | 12 |
| Coffee | MSMA | 93.23 | 0.6 | | 16 |
| Coffee | Oxyfluorfen | 42.46 | 4.3 | | 10 |
| Coffee | Pendimethalin | 32.13 | 0.3 | | 42 |
| Coffee | Pyraclostrobin | 17.71 | 3.2 | | 32 |
| Coffee | Pyridaben | 28.86 | 0.3 | | 14 |
| Coffee | Pyriproxyfen | 40.31 | 0.3 | | 16 |
| Coffee | Profenofos | 12.91 | 1.8 | | 70 |
| Coffee | Propargite | 26.17 | 7.8 | | 42 |
| Coffee | Propiconazole | 9.53 | 0.3 | | 12 |
| Coffee | Saflufenacil | 26.40 | 0.2 | | 10 |
| Coffee | Simazine | 64.10 | 0.9 | | 30 |
| Coffee | Sulfentrazone | 95.92 | 13.0 | | 12 |
| Coffee | Terbufos | 72.06 | 65.0 | | 40 |
| Coffee | Tetraconazole | 29.19 | 26.0 | | 14 |
| Coffee | Thiamethoxam | 14.58 | 2.6 | | 56 |
| Coffee | Thiophanate-methyl | 41.23 | 4.9 | | 30 |
| Coffee | Triadimenol | 89.55 | 2.6 | | 14 |
| Coffee | Zeta-cypermethrin | 12.26 | 15.6 | | 32 |
| Sugarcane | Alachlor | 20.61 | 0.5 | | 32 |
| Sugarcane | Ametryn | 12.78 | 0.0 | | 36 |
| Sugarcane | Aminopyralid | 100.00 | 0.0 | | 12 |
| Sugarcane | Asulam | 11.00 | 0.1 | | 10 |
| Sugarcane | Atrazine | 27.50 | 0.6 | | 50 |
| Sugarcane | Benfuracarb | 100.00 | 32.8 | | 16 |
| Sugarcane | Bispyribac | 29.09 | 0.5 | | 10 |
| Sugarcane | Carbosulfan | 52.02 | 6.6 | | 60 |
| Sugarcane | Cyclaniliprole | 10.16 | 0.0 | | 10 |
| Sugarcane | Mepiquat chloride | 100.00 | 0.1 | | 12 |
| Sugarcane | Fipronil | 13.38 | 7.4 | | 64 |
| Sugarcane | Flazasulfuron | 35.87 | 0.4 | | 15 |
| Sugarcane | Fluensulfone | 12.43 | 1.0 | | 18 |
| Sugarcane | Fluroxypyr-meptyl | 13.48 | 0.0 | | 12 |
| Sugarcane | Flutriafol | 10.57 | 1.5 | | 18 |
| Sugarcane | Glufosinate | 37.65 | 7.4 | | 56 |
| Sugarcane | Halosulfuron-methyl | 30.17 | 0.0 | | 10 |
| Sugarcane | Hexazinone | 100.00 | 0.1 | | 24 |
| Sugarcane | Isoxaflutole | 86.08 | 0.2 | | 12 |
| Sugarcane | MCPA | 70.48 | 0.0 | | 12 |
| Sugarcane | Mesotrion | 52.02 | 0.1 | | 20 |
| Sugarcane | Metribuzim | 13.24 | 0.4 | | 28 |
| Sugarcane | Simazine | 12.15 | 0.2 | | 20 |
| Sugarcane | Tebuthiuron | 100.00 | 0.7 | | 36 |
| Sugarcane | Terbuthylazine | 16.72 | 0.1 | | 30 |
| Sugarcane | Triclopyr butotyl | 21.54 | 0.1 | | 36 |
| Onion | Acephate | 11.44 | 1.8 | | 80 |
| Onion | Benalaxyl | 35.89 | 0.0 | | 15 |
| Onion | Benthiavalicarbisopropyl | 12.54 | 0.0 | | 10 |
| Onion | Captan | 10.87 | 1.4 | | 10 |
| Onion | Cymoxanil | 31.31 | 0.1 | | 12 |
| Onion | Dicloran | 100.00 | 0.7 | | 10 |
| Onion | Fenamidone | 12.95 | 0.0 | | 10 |
| Onion | Fenoxaprop-p-ethyl | 16.63 | 2.3 | | 10 |
| Onion | Mandipropamid | 37.31 | 0.2 | | 16 |
| Onion | Metalaxyl-m | 37.49 | 0.1 | | 12 |
| Onion | Metam | 100.00 | 21.3 | | 10 |
| Onion | Methiram | 9.54 | 0.7 | | 15 |
| Onion | Ioxynil octanoate | 100.00 | 0.1 | | 12 |
| Onion | Prometryn | 78.11 | 0.0 | | 10 |
| Onion | Propineb | 11.20 | 4.3 | | 45 |
| Citrus | 2,4-D | 38.53 | 5.5 | | 105 |
| Citrus | Acephate | 29.70 | 4.6 | | 120 |
| Citrus | Acrinathrin | 100.00 | 0.1 | | 15 |
| Citrus | Amitraz | 30.81 | 1.4 | | 18 |
| Citrus | Azoxystrobin | 14.07 | 9.7 | | 12 |
| Citrus | Bistrifluron | 100.00 | 0.1 | | 12 |
| Citrus | Bromacil | 100.00 | 0.1 | | 12 |
| Citrus | Buprofezin | 36.16 | 0.8 | | 15 |
| Citrus | Captan | 10.58 | 1.4 | | 10 |
| Citrus | Carbendazim | 17.10 | 6.9 | | 60 |
| Citrus | Carfentrazone-ethyl | 21.09 | 0.0 | | 12 |
| Citrus | Cyantraniliprole | 80.61 | 1.4 | | 12 |
| Citrus | Cyflumetofen | 33.23 | 0.0 | | 10 |
| Citrus | Cyprodinil | 77.50 | 0.5 | | 12 |
| Citrus | Benzalkonium chloride | 91.60 | 2.8 | | 12 |
| Citrus | Chlorfenapyr | 14.59 | 0.5 | | 84 |
| Citrus | Chlorfluazuron | 16.80 | 0.6 | | 20 |
| Citrus | Chlorpyrifos | 28.66 | 5.5 | | 168 |
| Citrus | Clothianidin | 46.86 | 0.1 | | 28 |
| Citrus | Chromafenozide | 78.93 | 0.0 | | 12 |
| Citrus | Dimethoate | 58.76 | 27.6 | | 42 |
| Citrus | Spinetoram | 25.55 | 0.7 | | 10 |
| Citrus | Spiropidion | 18.79 | 0.1 | | 12 |
| Citrus | Etoxazole | 23.71 | 0.1 | | 10 |
| Citrus | Fenpyroximate | 25.91 | 1.4 | | 14 |
| Citrus | Flonicamid | 90.57 | 0.2 | | 12 |
| Citrus | Florylpicoxamid | 30.02 | 0.2 | | 12 |
| Citrus | Flubendiamide | 19.31 | 0.6 | | 12 |
| Citrus | Fludioxonil | 22.13 | 5.5 | | 12 |
| Citrus | Flufenoxuron | 20.06 | 0.1 | | 12 |
| Citrus | Fluopyram | 12.17 | 0.7 | | 10 |
| Citrus | Folpet | 59.20 | 2.8 | | 12 |
| Citrus | Phosmet | 65.19 | 5.5 | | 21 |
| Citrus | Hexythiazox | 100.00 | 0.9 | | 12 |
| Citrus | Imazalil | 62.64 | 4.6 | | 42 |
| Citrus | Indaziflam | 51.74 | 0.0 | | 12 |
| Citrus | Isocycloseram | 78.16 | 0.3 | | 12 |
| Citrus | Lufenuron | 23.91 | 0.7 | | 12 |
| Citrus | Metaflumizone | 52.09 | 1.8 | | 10 |
| Citrus | Milbemectin | 52.15 | 0.0 | | 15 |
| Citrus | Fenbutatin oxide | 100.00 | 1.8 | | 15 |
| Citrus | Pydiflumetofem | 35.84 | 0.2 | | 12 |
| Citrus | Pyridaben | 18.39 | 0.2 | | 14 |
| Citrus | Pyrimethanil | 87.94 | 0.7 | | 12 |
| Citrus | Pyriproxyfen | 42.81 | 0.3 | | 24 |
| Citrus | Pyroxasulfone | 60.59 | 0.3 | | 12 |
| Citrus | Propargite | 46.32 | 13.8 | | 63 |
| Citrus | Propiconazole | 10.12 | 0.3 | | 36 |
| Citrus | Sulfometuron-methyl | 100.00 | 0.0 | | 12 |
| Citrus | Tebufenozide | 78.93 | 0.7 | | 12 |
| Citrus | Teflubenzuron | 34.20 | 1.4 | | 12 |
| Citrus | Thiabendazole | 45.50 | 2.8 | | 12 |
| Citrus | Tiafenacil | 100.00 | 0.1 | | 12 |
| Citrus | Thiophanate-methyl | 14.60 | 1.7 | | 45 |
| Citrus | Triflumuron | 48.29 | 2.0 | | 24 |
| Coconut | Diafenthiuron | 12.71 | 14.5 | | 24 |
| Coconut | Propargite | 10.88 | 2.61 | | 21 |
| Kale | Chlorfluazuron | 18.02 | 0.7 | | 10 |
| Bean | Abamectin | 41.83 | 4.8 | | 80 |
| Bean | Acephate | 30.68 | 4.8 | | 80 |
| Bean | Acifluorfen-sodium | 93.16 | 0.4 | | 14 |
| Bean | Amicarbazone | 98.01 | 8.6 | | 16 |
| Bean | Bentazone | 10.15 | 0.0 | | 12 |
| Bean | Emamectin benzoate | 27.29 | 5.7 | | 14 |
| Bean | Beta-cyfluthrin | 15.66 | 1.4 | | 16 |
| Bean | Bifenthrin | 19.66 | 7.1 | | 64 |
| Bean | Bixafen | 13.95 | 12.8 | | 16 |
| Bean | Boscalid | 56.48 | 0.7 | | 16 |
| Bean | Bromuconazole | 76.14 | 5.7 | | 12 |
| Bean | Buprofezin | 18.68 | 0.4 | | 10 |
| Bean | Captan | 21.87 | 2.9 | | 10 |
| Bean | Carbaryl | 78.71 | 47.5 | | 16 |
| Bean | Carbendazim | 70.65 | 28.5 | | 40 |
| Bean | Carboxin | 48.17 | 0.6 | | 10 |
| Bean | Cyfluthrin | 40.96 | 1.4 | | 18 |
| Bean | Cyromazine | 94.80 | 4.3 | | 20 |
| Bean | Clethodim | 33.74 | 25.7 | | 48 |
| Bean | Chlorfenapyr | 30.15 | 1.0 | | 56 |
| Bean | Chlorfluazuron | 17.36 | 0.6 | | 30 |
| Bean | Chlorothalonil | 20.47 | 19.0 | | 48 |
| Bean | Chlorpyrifos | 14.80 | 2.9 | | 112 |
| Bean | Clothianidin | 32.28 | 0.1 | | 42 |
| Bean | Deltamethrin | 12.48 | 10.0 | | 32 |
| Bean | Diafenthiuron | 28.64 | 28.5 | | 12 |
| Bean | Dicamba | 92.86 | 3.8 | | 10 |
| Bean | Diflubenzuron | 15.04 | 1.1 | | 30 |
| Bean | Dimoxystrobin | 100.00 | 4.8 | | 12 |
| Bean | Spinetoram | 26.40 | 0.7 | | 10 |
| Bean | Spinosad | 13.11 | 0.4 | | 12 |
| Bean | Spiromesifen | 16.98 | 1.0 | | 16 |
| Bean | Etofenprox | 15.04 | 4.8 | | 10 |
| Bean | Famoxadone | 90.66 | 14.3 | | 16 |
| Bean | Fenoxaprop-p-ethyl | 41.82 | 5.7 | | 10 |
| Bean | Fenpyrazamin | 93.16 | 0.1 | | 10 |
| Bean | Fenpyroximate | 16.06 | 0.9 | | 14 |
| Bean | Fipronil | 25.81 | 14.3 | | 64 |
| Bean | Fluazifop-p-butyl | 48.22 | 399.1 | | 12 |
| Bean | Fluazinam | 17.18 | 2.9 | | 30 |
| Bean | Flubendiamide | 34.92 | 1.2 | | 12 |
| Bean | Flumioxazin | 38.97 | 0.7 | | 36 |
| Bean | Fluopyram | 62.84 | 3.6 | | 10 |
| Bean | Flupyradifurone | 16.17 | 3.8 | | 14 |
| Bean | Fluroxypyr-meptyl | 78.01 | 0.0 | | 12 |
| Bean | Flutriafol | 20.38 | 2.9 | | 12 |
| Bean | Fomesafen | 78.43 | 4.8 | | 24 |
| Bean | Formetanate | 89.88 | 5.7 | | 36 |
| Bean | Gamma-cyhalothrin | 42.77 | 14.3 | | 10 |
| Bean | Glufosinate | 21.78 | 4.3 | | 56 |
| Bean | Halosulfuron-methyl | 69.83 | 0.1 | | 10 |
| Bean | Haloxyfop-p-methyl | 93.16 | 47.5 | | 42 |
| Bean | Fentin | 35.32 | 28.5 | | 18 |
| Bean | Imazethapyr | 33.45 | 0.1 | | 12 |
| Bean | Indoxacarb | 23.67 | 2.3 | | 14 |
| Bean | Malathion | 48.24 | 7.6 | | 48 |
| Bean | Mancozeb | 16.55 | 5.7 | | 50 |
| Bean | Metalaxyl-m | 15.08 | 0.0 | | 12 |
| Bean | Metconazole | 19.59 | 0.3 | | 12 |
| Bean | Methiram | 38.39 | 2.9 | | 15 |
| Bean | Methomyl | 11.87 | 0.7 | | 70 |
| Bean | Metominostrobin | 18.19 | 3.0 | | 16 |
| Bean | Oxicarboxin | 100.00 | 0.8 | | 10 |
| Bean | Pendimethalin | 35.26 | 0.3 | | 42 |
| Bean | Picoxystrobin | 9.53 | 0.1 | | 30 |
| Bean | Pyraflufen-ethyl | 94.08 | 0.0 | | 10 |
| Bean | Pyridaben | 31.66 | 0.3 | | 14 |
| Bean | Procymidone | 58.06 | 1.4 | | 30 |
| Bean | Profenofos | 20.24 | 2.9 | | 70 |
| Bean | Propiconazole | 10.45 | 0.4 | | 24 |
| Bean | Propineb | 45.07 | 17.1 | | 30 |
| Bean | Protioconazole | 42.09 | 17.1 | | 36 |
| Bean | Quizalofop-p-tefuryl | 91.51 | 1.1 | | 14 |
| Bean | Saflufenacil | 28.96 | 0.2 | | 10 |
| Bean | Sethoxydim | 96.46 | 2.0 | | 10 |
| Bean | S-metolachlor | 19.79 | 0.1 | | 40 |
| Bean | Sulfoxaflor | 87.54 | 19.2 | | 14 |
| Bean | Terbufos | 15.81 | 14.3 | | 40 |
| Bean | Tetraconazole | 12.81 | 11.4 | | 14 |
| Bean | Thiacloprid | 69.41 | 2.9 | | 14 |
| Bean | Thiodicarb | 45.77 | 1.0 | | 32 |
| Bean | Thiophanate-methyl | 15.08 | 1.8 | | 30 |
| Bean | Thiram | 21.56 | 1.7 | | 56 |
| Bean | Tolfenpyrad | 21.02 | 2.1 | | 15 |
| Bean | Trifloxystrobin | 22.87 | 1.9 | | 48 |
| Bean | Trifluralin | 29.36 | 0.6 | | 48 |
| Cowpea | Chlorothalonil | 20.47 | 19.0 | | 48 |
| Cowpea | Deltamethrin | 17.83 | 14.3 | | 32 |
| Cowpea | Diflubenzuron | 37.61 | 2.9 | | 30 |
| Cowpea | Fluazinam | 17.18 | 2.9 | | 30 |
| Cowpea | Flubendiamide | 34.92 | 1.2 | | 12 |
| Cowpea | Flupyradifurone | 16.17 | 3.8 | | 14 |
| Cowpea | Flutriafol | 20.38 | 2.9 | | 12 |
| Cowpea | Profenofos | 20.24 | 2.9 | | 70 |
| Cowpea | S-metolachlor | 19.79 | 0.1 | | 40 |
| Cowpea | Trifluralin | 29.36 | 0.6 | | 48 |
| Fava bean | Profenofos | 20.24 | 2.9 | | 70 |
| Fava bean | S-metolachlor | 19.79 | 0.1 | | 40 |
| Bean | Emamectin benzoate | 27.29 | 5.7 | | 14 |
| Bean | Beta-cyfluthrin | 15.66 | 1.4 | | 16 |
| Bean | Bifenthrin | 19.66 | 7.1 | | 64 |
| Bean | Buprofezin | 18.68 | 0.4 | | 10 |
| Bean | Clethodim | 33.74 | 25.7 | | 12 |
| Bean | Chlorfenapyr | 30.15 | 1.0 | | 56 |
| Bean | Chlorpyrifos | 14.80 | 2.9 | | 112 |
| Bean | Diafenthiuron | 28.64 | 28.5 | | 12 |
| Bean | Spinetoram | 26.40 | 0.7 | | 10 |
| Bean | Fenpyroximate | 16.06 | 0.9 | | 14 |
| Bean | Fipronil | 25.81 | 14.3 | | 64 |
| Bean | Fluazifop-p-butyl | 48.22 | 399.1 | | 12 |
| Bean | Glufosinate | 21.78 | 4.3 | | 56 |
| Bean | Imazethapyr | 33.45 | 0.1 | | 12 |
| Bean | Indoxacarb | 23.67 | 2.3 | | 14 |
| Bean | Mancozeb | 16.55 | 5.7 | | 50 |
| Bean | Metalaxyl-m | 15.08 | 0.0 | | 12 |
| Bean | Picoxystrobin | 9.53 | 0.1 | | 20 |
| Bean | Propiconazole | 10.45 | 0.4 | | 36 |
| Bean | Protioconazole | 42.09 | 17.1 | | 36 |
| Bean | Trifloxystrobin | 22.87 | 1.9 | | 12 |
| Apple | Amitraz | 16.45 | 0.7 | | 12 |
| Apple | Bixafen | 12.01 | 11.1 | | 16 |
| Apple | Captan | 28.26 | 3.7 | | 10 |
| Apple | Carbaryl | 16.27 | 9.8 | | 16 |
| Apple | Cyclaniliprole | 12.15 | 0.0 | | 10 |
| Apple | Cyflumetofen | 35.48 | 0.0 | | 10 |
| Apple | Dimethoate | 31.37 | 14.7 | | 28 |
| Apple | Dithianon | 100.00 | 2.9 | | 12 |
| Apple | Dodine | 100.00 | 7.4 | | 14 |
| Apple | Etoxazole | 25.32 | 0.1 | | 15 |
| Apple | Fenitrothion | 16.41 | 1.5 | | 48 |
| Apple | Fenpyroximate | 13.83 | 0.7 | | 14 |
| Apple | Fluazinam | 44.40 | 7.4 | | 30 |
| Apple | Flufenoxuron | 53.55 | 0.4 | | 12 |
| Apple | Folpet | 31.61 | 1.5 | | 12 |
| Apple | Phosmet | 34.81 | 2.9 | | 14 |
| Apple | Imazalil | 13.38 | 1.0 | | 28 |
| Apple | Iprodione | 10.57 | 1.2 | | 12 |
| Apple | Lufenuron | 15.32 | 0.4 | | 12 |
| Apple | Metaflumizone | 9.73 | 0.3 | | 10 |
| Apple | Metamitron | 100.00 | 0.0 | | 12 |
| Apple | Methidathion | 100.00 | 0.3 | | 20 |
| Apple | Methiram | 13.23 | 1.0 | | 45 |
| Apple | Procymidone | 12.00 | 0.3 | | 30 |
| Apple | Propineb | 15.53 | 5.9 | | 30 |
| Apple | Triflumizole | 37.42 | 0.0 | | 14 |
| Papaya | Abamectin | 12.06 | 1.3 | | 40 |
| Cassava | Prometryn | 21.89 | 0.0 | | 10 |
| Watermelon | Fenamidone | 13.21 | 0.0 | | 15 |
| Watermelon | Mandipropamid | 19.04 | 0.1 | | 16 |
| Watermelon | Pymetrozine | 27.00 | 0.1 | | 18 |
| Watermelon | Pyriofenone | 100.00 | 0.0 | | 10 |
| Watermelon | Triflumizole | 36.74 | 0.0 | | 14 |
| Corn | Alachlor | 20.53 | 0.5 | | 16 |
| Corn | Atrazine | 13.70 | 0.3 | | 25 |
| Corn | Carbosulfan | 25.90 | 3.3 | | 30 |
| Corn | Fenitrothion | 54.67 | 4.9 | | 24 |
| Corn | Mesotrion | 25.90 | 0.0 | | 20 |
| Corn | Permethrin | 21.22 | 1.5 | | 16 |
| Corn | Terbuthylazine | 83.28 | 0.6 | | 15 |
| Cabbage | Chlorfluazuron | 14.61 | 0.5 | | 20 |
| Cabbage | Methomyl | 13.23 | 0.9 | | 34 |
| Soybean | Carbosulfan | 22.08 | 2.8 | | 60 |
| Soybean | Cartap hydrochloride | 37.08 | 0.0 | | 18 |
| Soybean | Chlorimuron-ethyl | 26.68 | 0.0 | | 10 |
| Soybean | Fenpropidin | 29.13 | 0.0 | | 12 |
| Soybean | Glyphosate | 32.50 | 0.4 | | 60 |
| Soybean | Glufosinate | 10.66 | 2.1 | | 56 |
| Soybean | Lactofen | 100.00 | 0.1 | | 10 |
| Soybean | Linuron | 45.80 | 7.0 | | 12 |
| Soybean | MCPA | 14.96 | 0.0 | | 12 |
| Soybean | Mesotrion | 22.08 | 0.0 | | 10 |
| Soybean | Protioconazole | 10.30 | 4.2 | | 36 |
| Soybean | S-metolachlor | 23.24 | 0.2 | | 40 |
| Tomato | Acibenzolar-s-methyl | 17.03 | 0.1 | | 12 |
| Tomato | Afidopyropen | 55.18 | 0.0 | | 12 |
| Tomato | Alanycarb | 100.00 | 0.2 | | 21 |
| Tomato | Benalaxyl | 18.65 | 0.0 | | 10 |
| Tomato | Benthiavalicarbisopropyl | 65.18 | 0.1 | | 10 |
| Tomato | Buprofezin | 16.09 | 0.4 | | 10 |
| Tomato | Cymoxanil | 16.28 | 0.1 | | 12 |
| Tomato | Cyprodinil | 12.41 | 0.1 | | 12 |
| Tomato | Chlorfenapyr | 11.69 | 0.4 | | 56 |
| Tomato | Chlorfluazuron | 22.43 | 0.7 | | 10 |
| Tomato | Cartap hydrochloride | 13.06 | 0.0 | | 12 |
| Tomato | Kresoxim-methyl | 13.30 | 0.0 | | 12 |
| Tomato | Chromafenozide | 21.07 | 0.0 | | 12 |
| Tomato | Dimethomorph | 87.24 | 0.0 | | 12 |
| Tomato | Spiropidion | 20.07 | 0.1 | | 12 |
| Tomato | Ethephon | 11.44 | 1.0 | | 60 |
| Tomato | Fenamidone | 67.28 | 0.1 | | 10 |
| Tomato | Florylpicoxamid | 12.02 | 0.1 | | 12 |
| Tomato | Flufenoxuron | 21.42 | 0.1 | | 18 |
| Tomato | Fluopicolide | 36.96 | 0.0 | | 12 |
| Tomato | Isocycloseram | 15.65 | 0.1 | | 12 |
| Tomato | Isofetamid | 89.14 | 0.0 | | 12 |
| Tomato | Mandestrobin | 94.26 | 0.0 | | 12 |
| Tomato | Mandipropamid | 15.51 | 0.1 | | 24 |
| Tomato | Metam-sodium | 45.08 | 14.7 | | 24 |
| Tomato | Methoxyfenozide | 80.41 | 0.0 | | 12 |
| Tomato | Milbemectin | 13.92 | 0.0 | | 15 |
| Tomato | Paclobutrazol | 100.00 | 0.0 | | 18 |
| Tomato | Pymetrozine | 55.00 | 0.3 | | 12 |
| Tomato | Profenofos | 10.46 | 1.5 | | 70 |
| Tomato | Propamocarb | 71.12 | 0.2 | | 12 |
| Tomato | Tebufenozide | 21.07 | 0.2 | | 12 |
| Tomato | Tolfenpyrad | 72.42 | 7.4 | | 10 |
| Tomato | Ametoctradin | 29.10 | 0.0 | | 12 |
| Wheat | Cartap hydrochloride | 18.04 | 0.0 | | 12 |
| Wheat | Fenitrothion | 22.68 | 2.0 | | 48 |
| Wheat | Fenpropidin | 70.87 | 0.1 | | 12 |
| Wheat | MCPA | 14.56 | 0.0 | | 12 |
| Wheat | Pinoxaden | 100.00 | 0.0 | | 10 |
| Wheat | Triflumizole | 25.85 | 0.0 | | 14 |
| Wheat | Triflumuron | 17.81 | 0.7 | | 16 |
| Wheat | Triticonazole | 100.00 | 0.1 | | 10 |
